# Factors influencing the spatial distributions of river microbial communities at the watershed scale: a case study involving the Wuding River Basin

**DOI:** 10.3389/fmicb.2025.1667966

**Published:** 2025-11-11

**Authors:** Nan Xue, Manhong Xia, Bo Hu, Xinru Gong, Zhoufeng Wang, Xiaohong Zhao

**Affiliations:** 1 Key Laboratory of Subsurface Hydrology and Ecological Effect in Arid Region of the Ministry of Education, Chang’an University, Xi’an, China; 2 School of Water and Environment, Chang’an University, Xi’an, China; 3 Key Laboratory of Eco-hydrology and Water Security in Arid and Semi-arid Regions of Ministry of Water Resources, Chang’an University, Xi’an, China; 4 School of Civil Engineering, Chang’an University, Xi’an, China

**Keywords:** Wuding River Basin, gene sequencing, microbial diversity, environmental factors, geomorphological changes

## Abstract

Microbial communities regulate water quality and biogeochemical cycling in rivers, but their responses to geomorphological factors remain unclear. Water samples were collected in August 2024 (summer wet season) from the Wuding River, and metagenomic sequencing was used to investigate microbial community changes and the influences of geomorphological factors. Environment (nutrients, etc.,) exhibited significant spatial heterogeneity with temperature (*p* < 0.01), total organic carbon (TOC, *p* < 0.001), dissolved organic carbon (DOC, *p* < 0.001), chemical oxygen demand (COD, *p* < 0.05), total phosphorus (TP, *p* < 0.001) and suspended solids (SS, *p* < 0.001), which were significantly higher downstream than upstream. *Pseudomonadota*, *Cyanobacteriota*, and *Actinomycetota* were the most important microbial phyla, and *Cyanobacteriota* (*p* = 0.016) was significantly more abundant upstream than downstream. The linear discriminant analysis effect size (LEfSe) revealed 8 and 10 biomarkers upstream and downstream, respectively. Upstream microbial communities were adapted to oligotrophic and high-light environments, whereas heterotrophic, carbon-metabolizing communities occurred downstream. Significantly higher ACE (*p* < 0.05), Chao1 (*p* < 0.05), Shannon (*p* < 0.001), and Pielou’s evenness (*p* < 0.001) indices were observed downstream than upstream. The relative abundance of genes associated with carbon cycling (the methane metabolism pathway, TCA cycle, and rTCA cycle) was greater downstream than upstream, as was the relative abundance of nitrogen functional genes. Elevation affected the upstream microbial communities, whereas temperature, TP, TOC, and nitrate nitrogen (NO_3_-N) affected the downstream communities. The results improve our understanding of how geomorphology drives the environmental factors and then governs the microbial community and their carbon and nitrogen cycling pathways.

## Highlights

- Geomorphological types are a key driver of the water quality differences observed between the upstream and downstream areas.
- Significant upstream and downstream differences in microbial alpha diversity are observed.
- *Cyanobacteriota* exhibits greater relative abundance in the upstream.
- Most carbon and nitrogen cycle pathways are more abundant downstream than upstream.

## 1 Introduction

Microorganisms are irreplaceable in core ecological processes such as substance circulation, energy flow, and pollutant degradation; additionally, microorganisms exhibit application potential in the fields of biological control, industrial production (food, pharmaceuticals, and biofuels), and environmental remediation (Alabbosh, 2025; Hui et al., 2023). In river ecosystems, microbial communities are biological barriers against wastewater pollution and act as key regulators of the physicochemical balance and ecological stability of water bodies (Bian et al., 2024; Pang et al., 2024). Microbial functional activity directly affects the health of river ecosystems. Therefore, identifying the community characteristics and functional attributes of river microorganisms may inform our assessments of ecosystem health and support water resource protection and ecological security operations.

The environment affects the distribution of microbial communities and, to some extent, drives spatial variations in microbial diversity (Martiny et al., 2006). In recent decades, researchers have conducted numerous studies on the microbiology of water bodies, including the structures and compositions of microbial communities and their influences on ecosystems (Bagagnan et al., 2024; Chen et al., 2020; Fan et al., 2019). For example, Wu D. et al. (2024) reported that the spatial distribution of microbial communities in Yangcheng Lake and its inlet rivers was significantly correlated with environmental factors such as total nitrogen (TN). Zhang et al. (2022b) noted in their study of the Jialing River that chemical oxygen demand (COD) and ammonia nitrogen (NH_3_-N) exerted a significant influence on the diversity and composition of microbial communities in both upstream and downstream regions. Wang L. et al. (2018) analyzed the water—sediment dynamics in the Nanchong section of the Jialing River and its two urban tributaries, finding that environmental factors such as total phosphorus (TP), nitrate (NO_3_^–^) and metals (Zn and Fe) influenced alterations in their microbial community structures. However, changes in temperature, TP, total organic carbon (TOC), and nitrogen, as well as their effects on the structures and functions of river microbial communities at the watershed scale, have received less attention.

Amplicon and macrogenome sequencing are commonly used methods of microbial research in aquatic environments. With groundbreaking advancements in next-generation sequencing technologies, 16S/18S/ITS-based amplicon sequencing and metagenomic sequencing have emerged as cutting-edge approaches in riverine microbial research. For example, amplicon sequencing enables precise interrogation of specific genomic regions, revealing microbial community structure and rare species distributions (Valverde et al., 2021; Zhang et al., 2017). Metagenomic sequencing technology uses the genomes of microbial communities in specific environments to analyze the characteristics of microbial communities and identify the functional genes of microorganisms, thus overcoming traditional culture limitations (Ininbergs et al., 2015; Niegowska et al., 2021; Offiong et al., 2023). Although amplicon sequencing demonstrates high sensitivity and enables efficient microbial community composition profiling, its reliance on conserved marker genes constrains the resulting functional resolution, whereas metagenomic sequencing provides comprehensive insights into functional genes and metabolic pathways (Maki et al., 2019; Venbrux et al., 2023). Therefore, we selected metagenomic sequencing technology to study the microorganisms in the Wuding River Basin.

The Wuding River is a major primary tributary located in the middle reaches of the Yellow River and is the largest river in the Yulin region of Shaanxi Province, China (Tao et al., 2022; Xu et al., 2025). The health of the Wuding River watershed is critical for achieving ecological protection in the Yellow River Basin and for the sustainable development of the Yulin area. The upstream and downstream regions of the Wuding River Basin straddle the two geographical units of the Mu Us Sandland and the Loess Plateau, and its water–ecology–economy system exhibits cross-county coupling and coordination characteristics (Tao et al., 2022; Zhao et al., 2023). Xu et al. (2025) investigated runoff and sediment changes in the Wuding River Basin from 1956 to 2021 and their preliminary correlations with microbial communities, revealing that hydrological and sediment factors exert certain influences on microbial distribution. However, existing research remains largely confined to analyzing correlations between environmental factors and microbial communities throughout the year. During the wet season, when rainfall is concentrated and erosion rates are high, the research into how hydrological disturbances indirectly influence microbial community structure and functional potential by altering the spatiotemporal distribution of nutrients (e.g., carbon, nitrogen, and phosphorus) have yet to be systematically elucidated. Specifically, the starkly contrasting hydrodynamic conditions and sediment redistribution processes in different morphological units (such as the sandy upstream reaches versus the loess-dominated downstream areas) can profoundly reshape nutrient availability and microbial growth conditions, thus driving community succession and functional adaptation. To this end, focusing on this contrast between the upstream Mu Us Sandland and the downstream Loess Plateau of the Wuding River Basin during the summer (wet season), this research relies on hydrogeological parameters, aquatic nutrient indicators, and metagenomic data to clarify: (1) differences in water quality between the upper and lower reaches of the Wuding River Basin under varying geological and topographical influences, (2) under the influence of different geomorphic units, the differences in the compositional structure of microbial communities between the upstream and downstream areas and the key water quality driving factors, and (3) the functional potential of microbial communities in carbon and nitrogen cycling within the Wuding River Basin. The results improve our understanding of riverine microorganisms and their ecological impacts and provide a foundation for conducting ecological restoration in the Wuding River Basin and the Yellow River Basin.

## 2 Materials and methods

### 2.1 Study area and sampling

**FIGURE 1:**
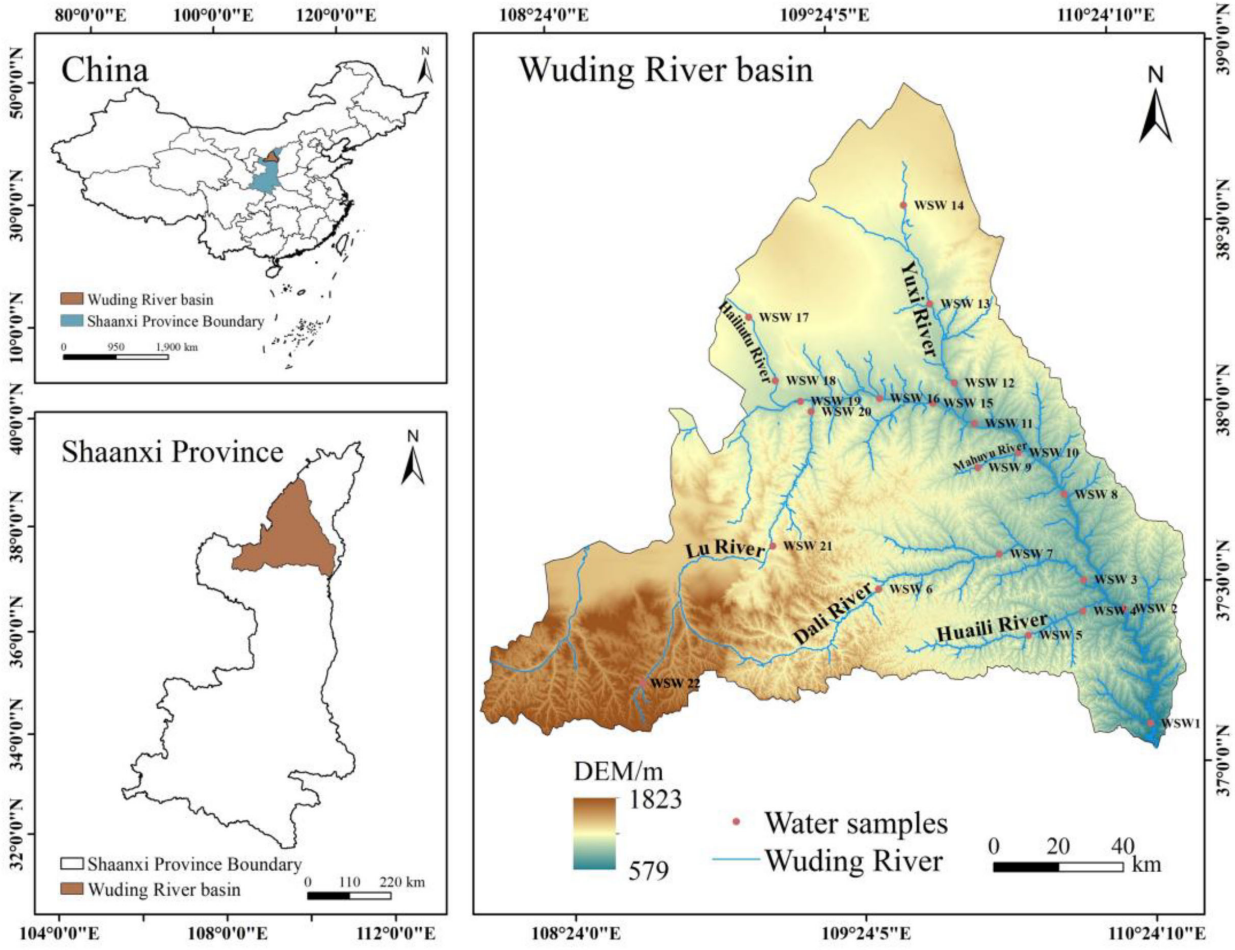
Sampling sites in the Wuding River Basin. Dots are water sampling points. The slender black line delineates the boundary of the Wuding River Basin in Shaanxi Province, encompassing both the main stream and its tributaries.

The primary basin of the Wuding River, a major middle-reach tributary of the Yellow River, lies within Yulin City, Shaanxi Province, China (108°27′39″ ∼ 110°34′22″E, 37°02′31″∼ 38°55′52″N). The Wuding River originates in the northern foothills of Baiyu Mountain in Dingbian County, Shaanxi Province, and stretches approximately 491 km in total length. It flows through the distinct geological landscapes of the Mu Us Sandland and the Loess Plateau. The Wuding River Basin encompasses several major tributary systems, including the Yuxi River, Lu River, Dali River, Huaining River, and Hailiutu River. With an average annual temperature of 9.5 °C and an average precipitation of 409.1 mm, the basin has a typical warm-temperate semiarid continental monsoon climate. Approximately 74% of the annual precipitation falls during summer and autumn (June–September, Figure 1). The upper reaches of the Wuding River traverse the southern margin of the Mu Us Sandland, characterized primarily by wind erosion. This section exhibits high permeability, low runoff yield, and the input of coarse-grained, nutrient-poor sandy sediments. In contrast, downstream lies the severely eroded the Loess Plateau, where hydrological processes predominate: loose Loess parent material, intense gully erosion and human activities collectively drive the sustained, high-intensity transport of fine-grained sediments and associated nutrients into rivers, creating a unique biogeochemical environment characterized by high sediment loads.

### 2.2 Sample collection and analysis

A total of 22 sampling sites (WSW1–WSW22) were deployed along the mainstem and tributaries of the Wuding River in August 2024 (Figure 1). Among them, sampling points WSW1–WSW11 represent the downstream river section, characterized by relatively strong flow velocities, elevated sediment loads, and comparatively significant anthropogenic impacts. In contrast, sampling points WSW12–WSW22 represent the upstream river section, exhibiting weaker hydrodynamic conditions, lower sediment transport, and lower population density. At each sampling site, 5 L of water was collected at a depth of 0.5 m. Subsequently, 3 L of every water sample was filtered through a 0.22 μm mixed fiber microporous membranes (Shanghai Xinya) and stored in a controlled environment at −80 °C for DNA extraction. A total of 2 L of every water sample was acidified to ensure sample preservation and the accuracy of subsequent physicochemical analyses in the laboratory. Each sampling point had three replicates.

A portable multiparameter water quality tester (HACH, HQ30d, United States) was used in the field to determine temperature, pH, DO, and electrical conductivity (EC) data. The concentration of suspended solids (SS, standard method: GB/T 11901-1989) was determined using the gravimetric method; TN (HJ 636-2012), TP (GB/T 11893-1989), ammonia nitrogen (NH_3_-N, HJ 535-2009), nitrate nitrogen (NO_3_-N, HJ/T 346-2007), and COD (HJ/T 399-2007, Supplementary Text 1) were measured with a DR6000 UV–visible spectrophotometer; TOC (HJ 501-2009) and dissolved organic carbon (DOC, HJ 501-2009) concentrations were quantified following standard methods using a Shimadzu TOC-L analyzer (Japan).

### 2.3 Microbial DNA extraction and sequencing

The cetyltrimethylammonium bromide (CTAB) method was used to extract microbial DNA from the water samples. Initially, 1,000 μl of CTAB lysate was pipetted into a 2.0 ml eppendorf (EP) tube, followed by the addition of lysozyme. The water samples were then introduced into the lysate, and the mixture was incubated in a 65 °C water bath for 2–3 h to facilitate lysis. Once lysis was complete, the samples were centrifuged, and the resulting supernatant was transferred to a test tube containing an equal volume of a phenol (pH 8.0)-chloroform-isoamyl alcohol mixture (25:24:1). After being mixed by inverting the tube repeatedly, the samples were centrifuged at 12,000 rpm for 10 min. Next, chloroform-isoamyl alcohol (24:1) was added to the new supernatant, which was then mixed by inversion and centrifuged again at 12,000 rpm for 10 min. The supernatant obtained from this step was moved to a 1.5 mL centrifuge tube, and isopropanol was added. After shaking the mixture, it was stored at −20 °C for 24 h to promote DNA precipitation. Subsequently, the mixture was centrifuged at 12,000 rpm for 10 min, and the supernatant was decanted carefully to avoid disturbing the precipitate. The precipitate was rinsed twice with 1 ml of 75% ethanol. After each rinse, the samples were centrifuged, and any residual liquid was removed by aspiration with a pipette tip. To dissolve the DNA, 50 μL of ddH_2_O was added, and if needed, the mixture was incubated at 55 °C–60 °C for 10 min to aid dissolution. RNA contamination was eliminated by adding 1 μl of RNase A to the tubes, which were then incubated at 37 °C for 15 min. Finally, the concentration and purity of the extracted DNA were determined using an Agilent 5,400 instrument.

DNA libraries were constructed using the Rapid Plus DNA Lib Prep Kit for Illumina (RK20208) library preparation following the protocol of the manufacturer. The libraries were then sequenced on an Illumina NovaSeq PE150 Sequencing Platform from Wekemo Tech Co., (Shenzhen, China).

### 2.4 Bioinformatics analysis

To ensure their reliability, the raw sequencing data were preprocessed with KNEAD Data software. Adapter sequences with quality scores below 20 and DNA sequences shorter than 50 bp were removed using Cutadapt. The DNA sequences were compared to the predicted genes utilizing Bowtie2 (Langmead and Salzberg, 2012). Finally, FastQC software was utilized to perform quality control on the reads. Taxonomic annotation of clean sequences was performed using Kraken2 with a proprietary database (Wekemo Tech Co.,). Actual relative species abundance was then estimated using Bracken. After undergoing quality control and dehosting, the clean sequences were aligned with the UniRef90 protein database using HUMAnN3 software based on Diamond. Following alignment against the UniRef90 and Kyoto Encyclopedia of Genes and Genomes (KEGG^1^) databases, gene abundances assigned to identical functional categories were summed. This aggregation produced annotated functional abundance tables for each database.

### 2.5 Statistical analysis

Data processing and visualization utilized IBM SPSS Statistics 27 (International Business Machines Corporation, Armonk, New York), Wekemo Bioincloud, and Origin 2024 (OriginLab, Northampton, MA). A *t*-test, Welch’s *t*-test, and a non-parametric Mann–Whitney *U*-test were used to estimate the differences between samples. The beta diversity of the upstream and downstream microbial communities was analyzed with non-metric multidimensional scaling (NMDS) and principal coordinate analysis (PCoA). The linear discriminant analysis effect size (LEfSe) was used to identify indicator microbial taxa that differed significantly between the upstream and downstream regions. A redundancy analysis (RDA) combined with Spearman correlations was used to explore the relationships between microbial communities and the environmental factors. Differences between the carbon and nitrogen functions of the upstream and downstream microorganisms were analyzed using the KEGG database with DiTing software (Xue et al., 2021).

## 3 Results and discussion

### 3.1 Physical and chemical characteristics of the Wuding River Basin

**FIGURE 2:**
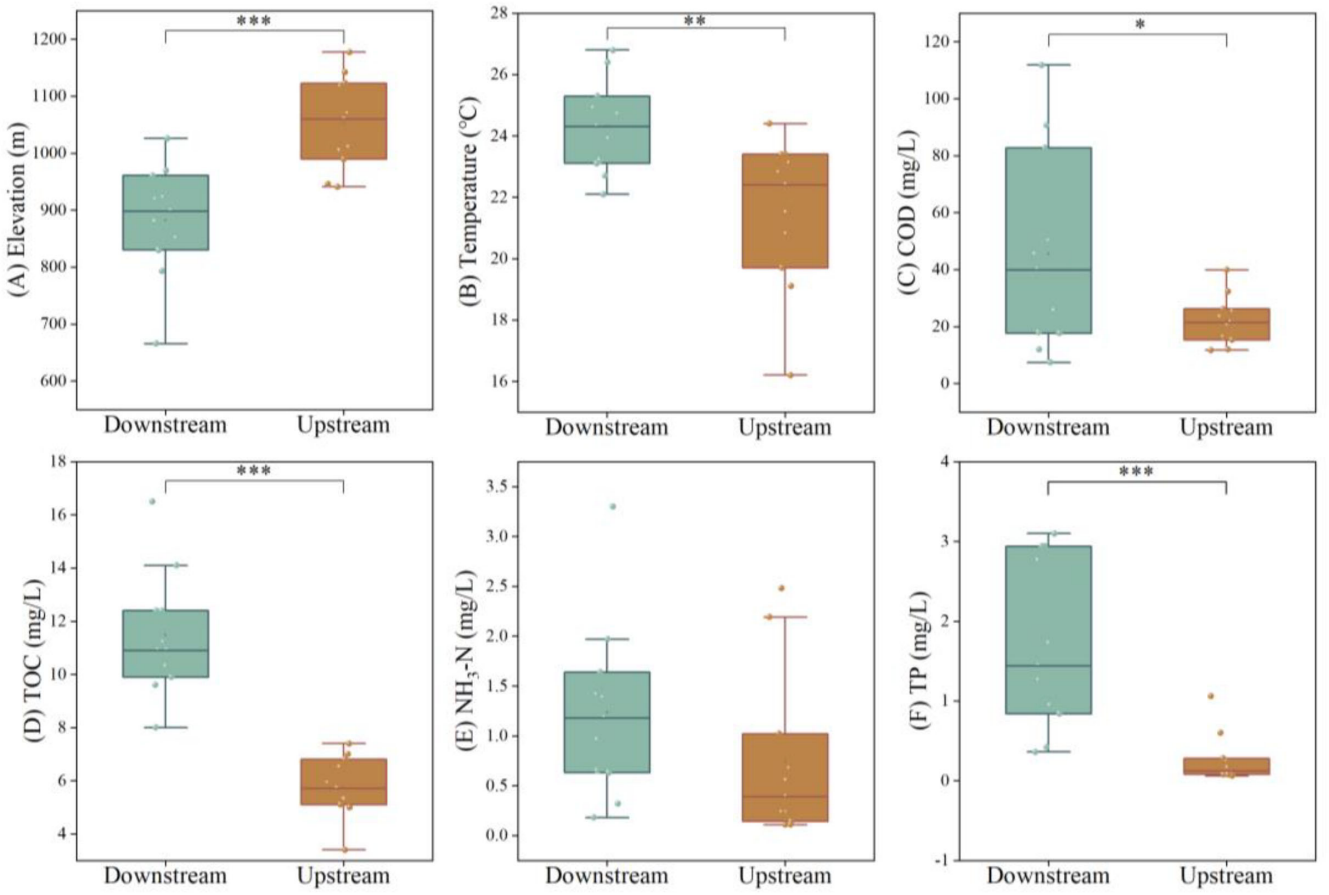
Analysis of physico-chemical properties [**(A)** Elevation; **(B)** Temperature; **(C)** COD; **(D)** TOC; **(E)** NH_3_-N; **(F)** TP] of water bodies upstream and downstream of the Wuding River Basin (*p < 0.05; **p < 0.01; ***p < 0.001).

Based on observational data from August 2024 (summer wet season), the hydrogeochemical parameters for the Wuding River Basin are presented in Table 1 and Supplementary Table 1. The pH of the water in the basin ranged from 8.01 to 8.75, representing a weakly alkaline environment; DO ranged from 57.70% to 95.70%, representing high oxygen content; and EC ranged from 461.00 to 1666.00 μs/cm. The mean values of TN, NH_3_-N, and NO_3_-N were greater downstream than upstream (Figure 2 and Supplementary Figure 1). The elevation in the Wuding River Basin ranged within 882.94 ± 93.54 m (mean ± standard deviation, downstream) and 1052.6 ± 76.89 m (upstream), the temperature ranged within 24.31 ± 1.43 °C (downstream) and 21.53 ± 2.3 °C (upstream), COD ranged within 45.55 ± 33.56 mg/L (downstream) and 22.17 ± 8.21 mg/L (upstream), and TP ranged within 1.7 ± 1.01 mg/L (downstream) and 0.26 ± 0.29 mg/L (upstream). The concentration of SS was variable and ranged within 46649.09 ± 73108.3 mg/L (downstream) and 450.45 ± 615.87 mg/L (upstream). From upstream to downstream, the concentrations of TOC and DOC increased gradually, with the TOC concentrations ranging within 11.47 ± 2.22 mg/L (downstream) and 5.76 ± 1.08 mg/L (upstream) and DOC concentrations ranging within 10.04 ± 2.39 mg/L (downstream) and 5.47 ± 1.06 mg/L (upstream).

**TABLE 1:** Hydrogeochemical parameters upstream and downstream of the Wuding River Basin.

Group	Downstream				Upstream			
	Min	Max	Mean	S. D.	Min	Max	Mean	S. D.
Elevation (m)	665.5	1025.9	882.94	93.54	940.9	1177.2	1052.6	76.89
Temp (°C)	22.1	26.8	24.31	1.43	16.2	24.4	21.53	2.3
pH	8.01	8.44	8.17	0.15	8.02	8.75	8.26	0.17
DO (%)	69	95.7	81.01	7.8	57.7	86.9	74.25	9.26
EC (μS/cm)	569	1,183	917.73	187.58	461	1,666	948.45	351.33
NH_3_-N (mg/L)	0.18	3.3	1.24	0.84	0.11	2.48	0.74	0.8
NO_3_-N (mg/L)	0.26	2.34	1.62	0.65	0.07	2.48	1.23	0.8
TN (mg/L)	5.5	11.35	7.65	2.01	4.28	11.8	7.64	3.16
TP (mg/L)	0.36	3.1	1.7	1.01	0.06	1.06	0.26	0.29
COD (mg/L)	7.39	111.79	45.55	33.56	11.73	39.86	22.17	8.21
SS (mg/L)	1,006	247,056	46649.09	73108.3	5	2,030	450.45	615.87
DOC (mg/L)	7.4	15.9	10.04	2.39	3.6	7.4	5.47	1.06
TOC (mg/L)	8	16.5	11.47	2.22	3.4	7.4	5.76	1.08

Temp, temperature; DO, dissolved oxygen; EC, electrical conductivity; NH_3_-N, ammonia nitrogen; NO_3_-N, nitrate nitrogen; TN, total nitrogen; TP, total phosphorus; COD, chemical oxygen demand; SS, suspended sediment; DOC, dissolved organic carbon; TOC, total organic carbon.

Significant differences in elevation, temperature, TOC, DOC, COD, TP, and SS were found between the upstream and downstream areas of the Wuding River Basin (Figure 2 and Supplementary Figure 1). Elevation was significantly higher in the upstream region (*p* < 0.001, *t*-test), while the downstream region exhibited significantly higher temperature (*p* < 0.01, *t*-test), TOC (*p* < 0.001, *t***-**test), DOC (*p* < 0.001, *t*-test), COD (*p* < 0.05, *t*-test), TP (*p* < 0.001, Mann-Whitney *U*-test), and SS (*p* < 0.001, Mann-Whitney *U*-test). Furthermore, TOC, DOC, and COD values showed greater variability in the downstream area compared to the upstream area. Nutrients (TN, NH_3_-N, NO_3_-N, COD, TOC, and DOC) were enriched downstream due to two main reasons: (1). the downstream is densely populated yet agricultural fertilization and domestic sewage discharge increase nitrogen and organic loads; and (2). along the direction of water flow, vegetation fallout and soil organic matter in the watershed are washed into the river, while higher water temperature accelerates the mineralization and decomposition of particulate organic matter (POC) in the sediment into DOC and CO_2_ by enhancing the microbial enzyme activity while releasing more oxidizable organic matter (Chen et al., 2023; Duan and Kaushal, 2013).

Additionally, this study revealed that the concentrations of SS and TP were significantly higher in the downstream than in the upstream (Table 1). This spatial heterogeneity is primarily driven by the combined effects of distinct soil erosion mechanisms and land use patterns between the Mu Us Sandland in the upstream and the Loess Plateau in the downstream.

Regarding SS concentrations, their concentration differences are closely related to regional surface composition and erosion dynamics. The upstream is primarily distributed within the Mu Us Sandland, where surface materials are dominated by coarse sand. The upstream features a loose structure, high permeability, poorly developed surface runoff, and limited sediment yield. SS concentrations predominantly exist as coarse particles with low transport capacity (Zhang and Zhang, 2022). In contrast, the downstream is widely covered by loessial soils rich in silt particles, which have poor erosion resistance. These soils are highly susceptible to rill erosion and sheet erosion under raindrop impact and runoff scouring, generating large amounts of fine-grained suspended sediment (Li et al., 2017). Furthermore, the high agricultural cultivation rate and relatively low vegetation coverage in the lower reaches further exacerbate soil erosion and the risk of SS concentrations input into the river (Zhao et al., 2023). A similar spatial differentiation pattern has been confirmed in studies of the Jialing River Basin, where SS concentrations in the mid-downstream regions with intensive agricultural activities were significantly higher than in the upstream natural vegetation-covered areas (Zhang et al., 2022b). Furthermore, the interpretation of variations in suspended solids should be extended to encompass multiple physical indicators such as particle size distribution, organic matter content, and mineral composition, and these should be comprehensively considered in subsequent research.

The distribution of TP concentrations also exhibits distinct regional input and transport characteristics. In the upstream, the soil is predominantly quartz sand, which has a weak capacity for phosphorus adsorption. Combined with significant wind erosion, particulate phosphorus is prone to long-distance transport (Šimanský et al., 2022). By contrast, the loess soils in downstream areas possess a certain capacity for adsorbing and retaining phosphorus. However, frequent agricultural activities (e.g., fertilizer application) have led to a high background phosphorus level in downstream areas. Under intense hydrological erosion, phosphorus readily enters the river adsorbed onto fine particles. It is noteworthy that although point source emissions (e.g., industrial wastewater) in the upstream contribute to part of the phosphorus load, their intensity is far lower than the non-point source dominated phosphorus input in the downstream (Tao et al., 2022). TP concentrations in the downstream section of the Wuding River basin were significantly higher than those in the upstream section (Figure 2), indirectly indicating that the migration pattern identified in this research is predominantly driven by non-point source pollution. This finding is consistent with the conclusions drawn from research on the Jialing River basin (Zhang et al., 2022b).

### 3.2 Diversity analysis and factors influencing the microbial communities in the Wuding River Basin

**FIGURE 3:**
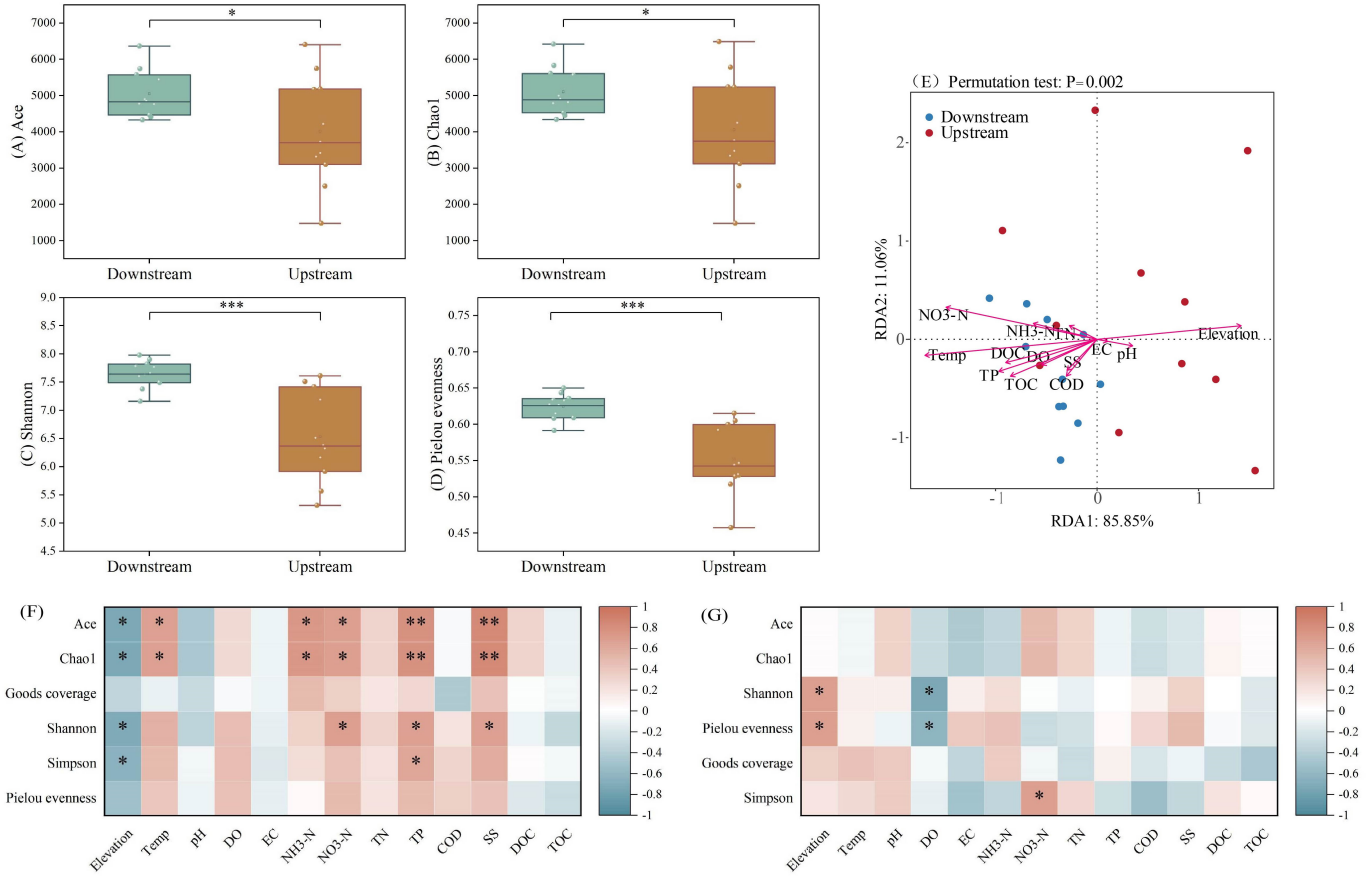
Alpha diversity [**(A)** ACE index; **(B)** Chao1 index; **(C)** Shannon index; **(D)** Pielou’s evenness index], redundancy analysis [RDA, **(E)**] analyses and correlation analysis [**(F)** Upstream; **(G)** Downstream] based on microorganisms from the Wuding River Basin (*, *p* < 0.05; **, *p* < 0.01; ***, *p* < 0.001).

The alpha diversity of the microbial communities in the upstream and downstream areas of the Wuding River Basin was analyzed (Figure 3). According to Welch’s *t*-test, the downstream values for the ACE (*p* < 0.05), Chao1 (*p* < 0.05), Shannon (*p* < 0.001), and Pielou’s evenness (*p* < 0.001) indices were significantly higher than those in the upstream reaches. This indicates that the richness, diversity, and evenness of the microbial community were all greater in the downstream region. Relationships between microbial alpha diversity and water quality indicators were explored via RDA (Figure 3E) and correlation heatmaps (Figures 3F, G). The primary two principal components, RDA1 and RDA2, accounted for 11.06% and 85.85% of the variation in microbial alpha diversity, respectively. In the upstream region, elevation, temperature and NO_3_-N drove microbial alpha diversity characteristics. Among these, ACE index and Chao1 index showed a negative correlation with altitude (*p* < 0.05) and a positive correlation with temperature and nitrate nitrogen (*p* < 0.05). In the downstream region, temperature, TP, and NO_3_-N drove the alpha diversity characteristics. Specifically, Shannon index was positively correlated with elevation (*p* < 0.05) and negatively correlated with DO (*p* < 0.05). These factors have been widely documented as drivers of riverine microorganisms (Shang et al., 2023; Wu Y. et al., 2024).

The Chao1, ACE, Shannon, and Pielou evenness indices were lower upstream than downstream, which may have been due to differences between the physicochemical properties of water quality in the upstream and downstream areas of the Wuding River Basin. First, higher downstream temperatures may have caused microbial activity and species richness to increase (Chen et al., 2023) and resulted in greater downstream microbial diversity. Second, higher downstream concentrations of TOC and DOC may have contributed to increased microbial diversity and abundance by providing nutrient substrates, regulating metabolic pathways, and promoting community differentiation (Rutere et al., 2020; Zhang et al., 2022a). In addition, the amount of industrial wastewater discharge in the upstream area of the basin was high (Tao et al., 2022; Zheng et al., 2024), and pollutants may decrease the microbial diversity in this area (Pascual-Benito et al., 2020; Wang et al., 2022). These combined factors (differences between the upstream and downstream physicochemical properties and anthropogenic impacts) accounted for the higher downstream microbial abundance, diversity, and evenness levels in the Wuding River Basin.

The beta diversity of the microbial communities in in the upstream and downstream areas of the Wuding River Basin was analyzed using NMDS and PCoA (Supplementary Figure 2). In the PCoA, Axis.1 explained 35.06% of the variance, and Axis.2 explained 15.39% of the variance; the total cumulative explanation of 50.45% of the variance indicates that the first two principal coordinates better reflect the overall differences between upstream and downstream areas. PERMANOVA revealed that the microbial community composition differed significantly in the upstream and downstream areas (*P* = 0.001) and that the upstream and downstream locations significantly explained the variation exhibited by the microbial community composition (*R*^2^ = 20.5%). The downstream sampling sites were more concentrated and the upstream sampling sites were more dispersed, which suggests that there was less variation in the microbial communities downstream than upstream. NMDS analyses supported this conclusion. This may be due to the fact that the remarkable variability in the beta diversity of microbial communities upstream and downstream of the Wuding River Basin stems mainly from its high environmental heterogeneity (Figure 2) and possible dispersal limitations (Soininen, 2010; Vellend, 2010). This heterogeneity leads to strong and diverse environmental filtering (Besemer et al., 2013), resulting in the formation of unique communities adapted to localized conditions at different sites.

### 3.3 Analysis of the water microbial community structure in the Wuding River

**FIGURE 4:**
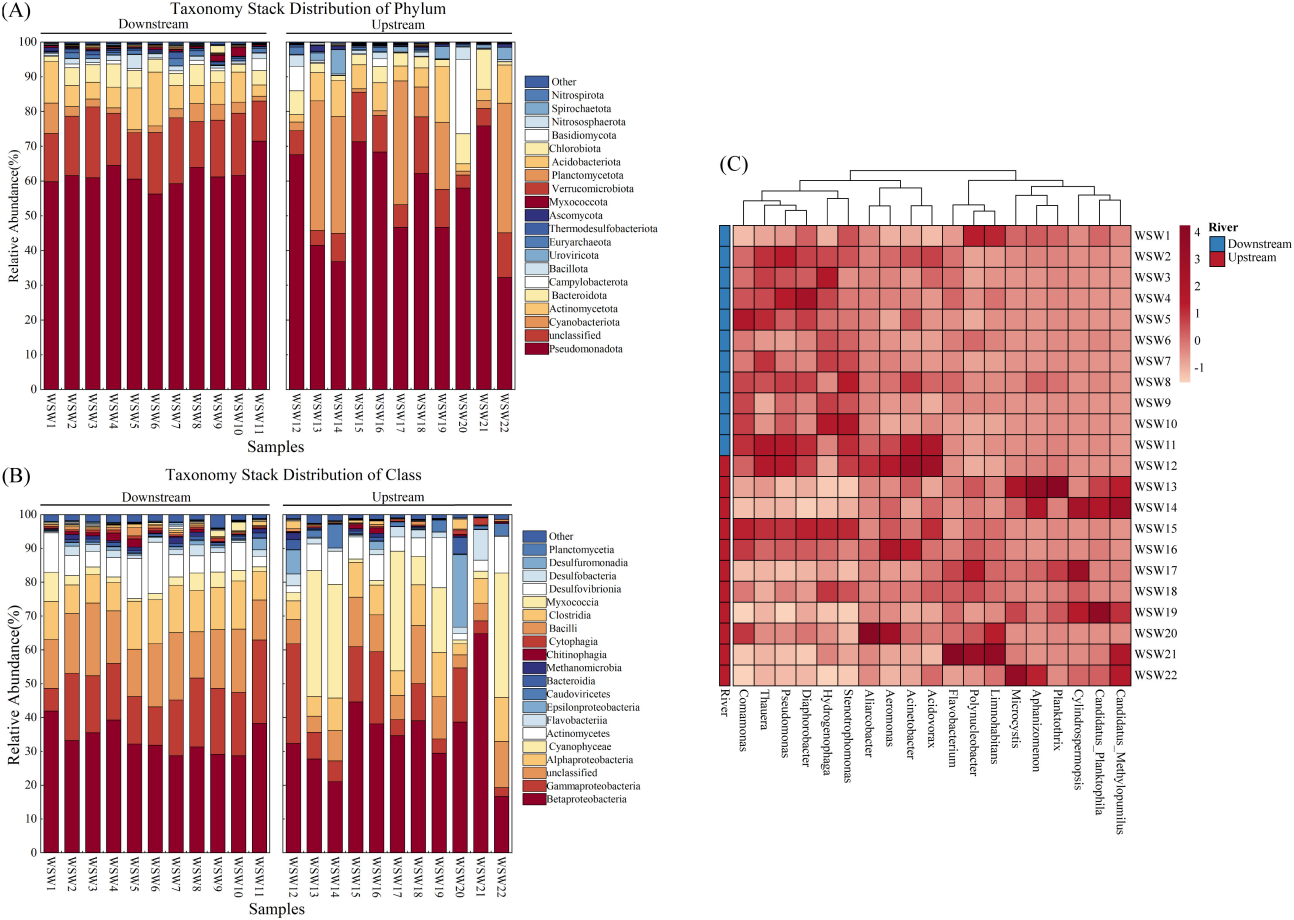
Analysis of microbial community structure in the Wuding River Basin. **(A)** Phylum level; **(B)** class level; **(C)** genus level.

Water microbial samples from the Wuding River Basin yielded a total of 92 phyla, 196 classes, 382 orders, 835 families, and 2,425 genera. Upstream samples contained 86 phyla, 186 classes, 362 orders, 773 families, and 2,225 genera, while downstream samples harbored 89 phyla, 190 classes, 366 orders, 797 families, and 2,296 genera. The microbial community structures in the upstream and downstream areas of the watershed were analyzed in detail at the phylum, class, and genus levels. We observed that *Pseudomonadota* was the most abundant phylum and varied from 32.25%–75.88% to 56.29%–71.51% in the upstream and downstream areas, respectively. Other dominant phyla included *Cyanobacteriota*, *Actinomycetota*, *Bacteroidota*, *Campylobacterota*, *Bacillota*, and *Uroviricota* (Figure 4A). Similar microbial community structures have been reported in the Yellow River (Ningxia River section) and the Seine River (Bagagnan et al., 2024; Wu D. et al., 2024; Zhao et al., 2023). At the class level, *Betaproteobacteria* (28.72%–41.93%), *Gammaproteobacteria* (6.70%–24.70%), *Alphaproteobacteria* (8.41%–14.23%), and *Cyanophyceae* (0.79%–8.64%) had higher relative downstream abundances, and *Betaproteobacteria* (16.66%–64.83%), *Cyanophyceae* (0.99%–37.24%), *Gammaproteobacteria* (2.63%–29.43%), and *Alphaproteobacteria* (12.97%–3.28%) had higher relative upstream abundances (Figure 4B). At the genus level, *Polynucleobacter* was the most abundant genus in the upstream and downstream areas of the basin, with relative abundances of 0.81%–30.92% and 1.12%–17.40%, respectively. In addition, *Limnohabitans* (0.51%–20.61%) and *Cylindrospermops is* (0.02%–18.12%) had higher relative upstream abundances, and *Pseudomonas* (2.67%–8.33%) and *Diaphorobacter* (2.44%–6.95%) had higher relative downstream abundances (Figure 4C).

Phylum level analysis shows that *Pseudomonadota* (*p* = 0.166, *t*-test) and *Actinomycetota* (*p* = 0.65, *t*-test) were not significantly different between the upstream and downstream areas. However, the relative abundance of *Cyanobacteriota* was greater upstream than downstream (*p* = 0.016, *t*-test). *Cyanobacteriota* play important roles in the carbon, nitrogen, and sulfur cycles. We observed that the relative abundance of *Cyanobacteriota* increased significantly under higher elevation and light conditions in the upper reaches of the Wuding River Basin. The concentration of SS upstream was lower than that downstream, which was in the Loess Plateau, and the upstream water clarity may have contributed to the greater relative abundance of *Cyanobacteriota upstream* (Klatt et al., 2020; Konrad et al., 2023; Tan et al., 2024). *Alphaproteobacteria (p* = 0.041, *t-*test) showed significant variability between upstream and downstream in the class level. *Alphaproteobacteria* can influence the biogeochemical transformations of elements through denitrification processes (e.g., *nosZ* gene-mediated nitrate reduction) in the nitrogen cycle (Bandekar et al., 2023; Yang et al., 2024) and sulfur oxidation processes in the sulfur cycle (Ghosh and Dam, 2009; Yang et al., 2024), and their representative genera (e.g., *Rhodopseudomonas* and *Bradyrhizobium*) may play key roles in N_2_O emissions (Bandekar et al., 2023). At the genus level, *Pseudomonas* (*p* = 0.027, *t*-test) showed significant differences between upstream and downstream, and the relative abundance of Pseudomonas was significantly higher downstream than upstream. *Pseudomonas* influences the nitrogen and sulfur cycles through processes such as nitrogen fixation (Busquets et al., 2013), denitrification (Zheng X. et al., 2023), and sulfur reduction (Musialowski et al., 2023). In this study, upstream and downstream differences between microbial community structures were observed at the phylum, class, and genus levels. Therefore, the upstream and downstream environments of the Wuding River Basin influenced the microbial community structure.

**FIGURE 5:**
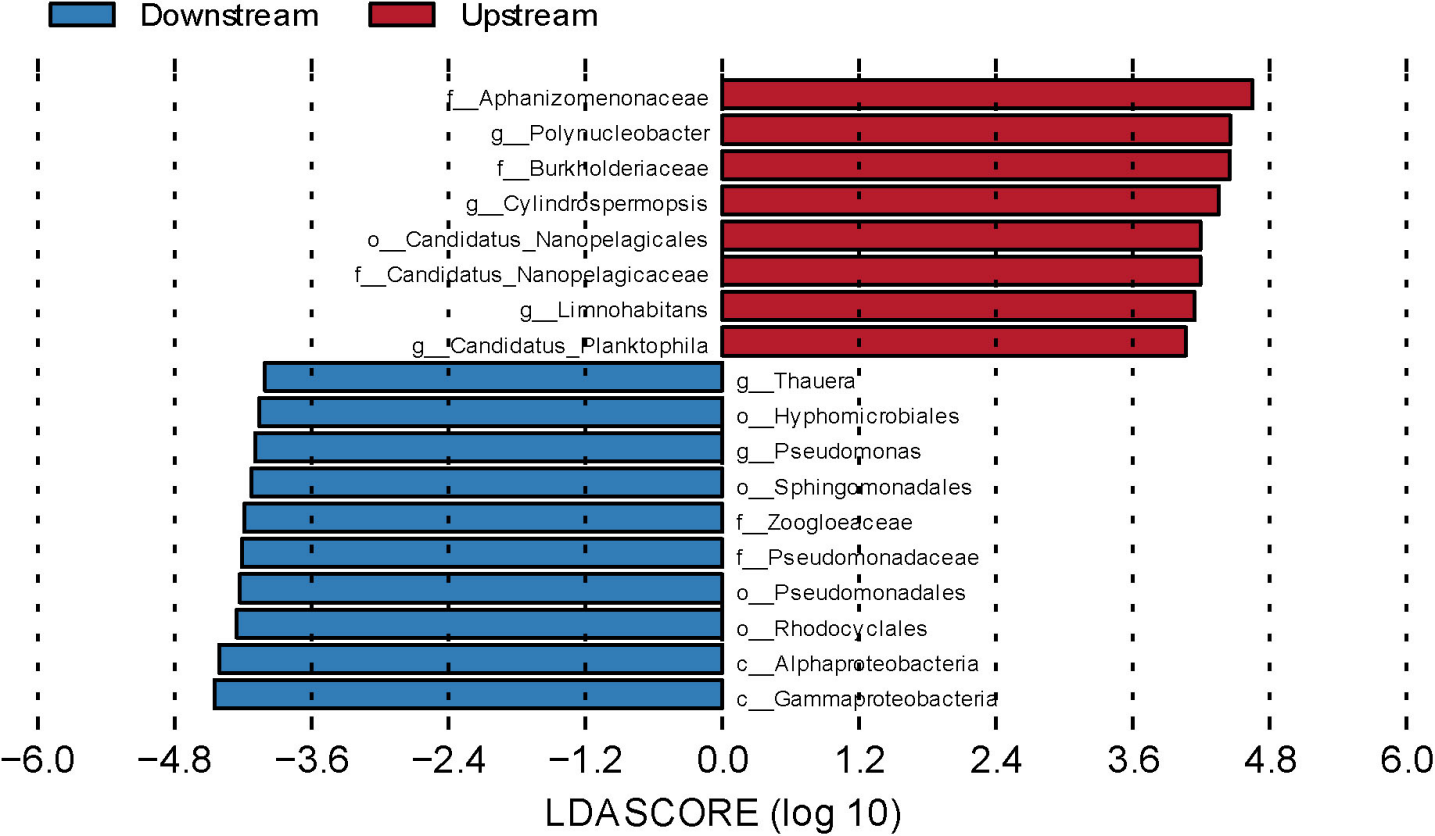
Linear discriminant analysis effect size (LEfSe) analysis of water microbial communities in upstream and downstream samples. Differentially abundant taxa, the histogram length represents the impact of different species [linear discriminant analysis (LDA) score = 4].

To explore the species differences between the upstream and downstream regions at spatial and temporal scales, we used LEfSe to identify microbial community taxa, which in turn revealed their marker microorganisms. Linear discriminant analysis (LDA > 4) was performed to identify different flora (Figure 5 and Supplementary Figure 3). The results revealed eight biomarkers upstream, including *Polynucleobacter*, *Candidatus Planktophila*, and *Limnohabitans* at the genus level and the order *Candidatus Nanopelagicales*. Ten biomarkers were identified downstream, including *Thauera* and *Pseudomonas* at the genus level and *Alphaproteobacteria* and *Gammaproteobacteria* at the class level. These biomarkers were the main causes of significant upstream and downstream variability.

Among the upstream biomarkers, *Candidatus Planktophila* belongs to *Actinomycetota* and is characteristic of inorganic nitrogen pollution (Yan et al., 2021), as this genus has adapted to nutrient-poor freshwater environments (Vincent et al., 2022) such as rivers with low TP and DOC levels. Moreover, TP and DOC were significantly lower upstream than downstream, which may be why *Candidatus Planktophila* is as a key upstream indicator species. *Candidatus Nanopelagicales* is also suited to survival in nutrient-poor freshwater environments. This order contains microorganisms that are more obviously nitrogen limited and can play a complementary role in the nitrogen cycle (Neuenschwander et al., 2018). *Limnohabitans*, a genus containing aerobic, anaerobic, and photoheterotrophic species, is involved in the carbon and nitrogen cycles and has a symbiotic relationship with *Polynucleobacter*, which further influences the ecological functioning of water bodies (Kasalický et al., 2018). Among the downstream biomarkers, *Pseudomonas* has the ability to degrade a wide range of pollutants, such as hydrocarbons, pesticides, and heavy metals (Song et al., 2025; Wang X. et al., 2018). *Thauera* can degrade pollutants, including aromatic compounds, and remove carbon, nitrogen, and phosphorus from wastewater (Ren et al., 2021). We observed that the TOC, DOC, and COD levels were significantly higher downstream than upstream, which may explain why *Pseudomonas* and *Thauera* were key downstream indicators. Overall, the upstream indicators were dominated by microbial communities that were suited to nutrient-poor and high-light environments, whereas the downstream indicators were dominated by complex, heterotrophic, carbon metabolism-related microbial communities. These findings align with the different ecological conditions in the upstream and downstream areas of the Wuding River Basin.

### 3.4 Correlations between water the microbial community structures and environmental factors in the Wuding River Basin

**FIGURE 6:**
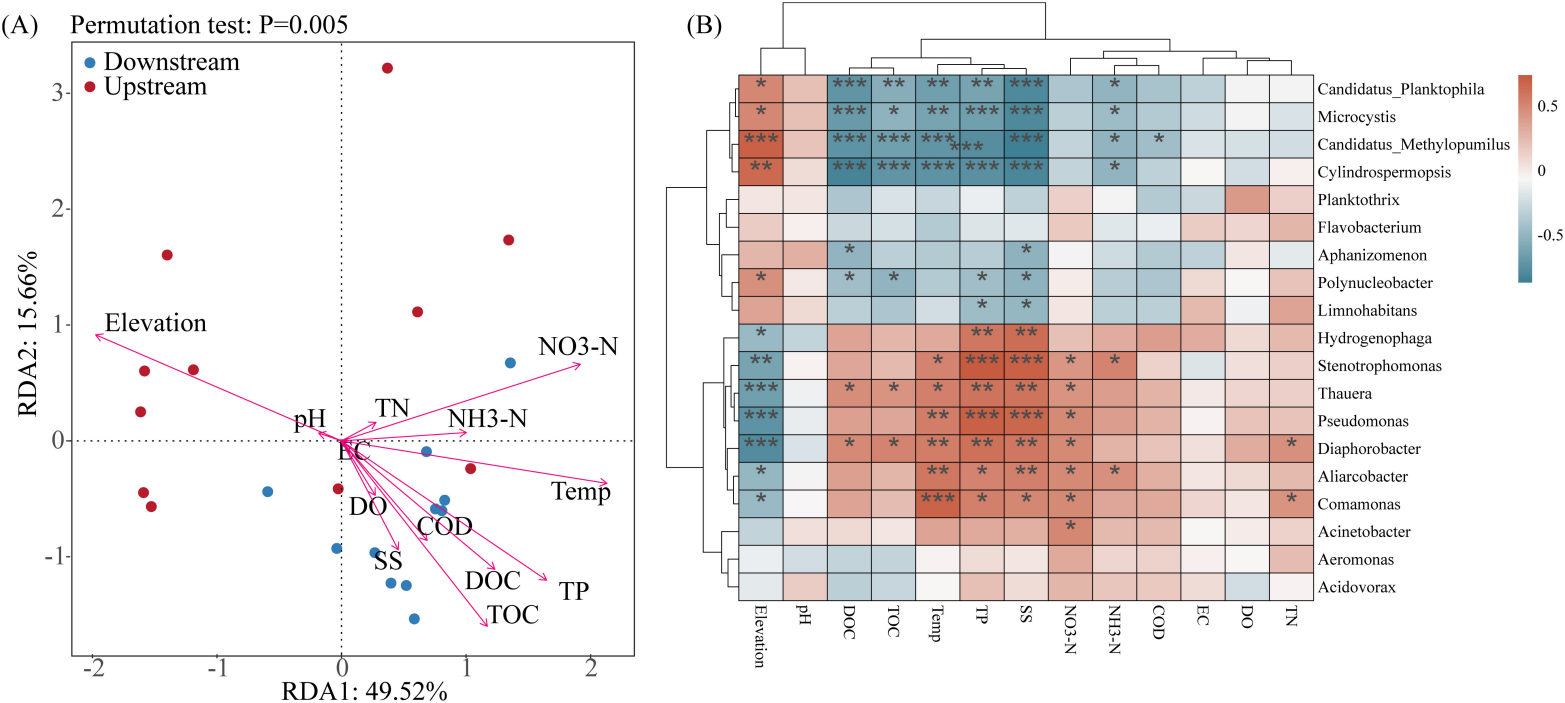
Environmental factors affecting microbial communities in the Wuding River Basin. **(A)** Redundancy analysis (RDA) analysis at genus level. **(B)** Spearman correlation heatmap analysis between environmental factors and genus level (*p < 0.05; **p < 0.01; ***p < 0.001).

Aquatic ecosystems are susceptible to environmental perturbations, and the diversity and structures of microbial communities are closely related to environmental changes. In this study, the relationships between environmental factors and the dominant microbial groups at the genus level were analyzed using RDA combined with Spearman’s correlation analysis. The first two principal components of the RDA (RDA1 and RDA2) explained 49.52% and 15.66%, respectively, of the variance in the structure of the microbial communities (Figure 6A). At the genus level, elevation was the main factor that affected the upstream distribution of the microbial communities, and temperature, TP, TOC, and NO_3_-N were the main factors that affected the downstream distribution of the microbial communities. These findings were similar to the results of the RDA conducted for microbial alpha diversity. A Spearman correlation analysis between microbial abundance (genus level) and environmental factors (Figure 6B) revealed that some of the dominant microbial communities in the basin were significantly correlated with elevation, TOC, DOC, temperature, TP, SS, NH_3_-N, and NO_3_-N.

Environmental factors can lead to changes in microbial communities (Wang et al., 2025). Gao et al. (2024) reported that temperature, EC, and NH_4_^+^-N affected the structures of the microbial communities in the water and sediment of the Yellow River. Bagagnan et al. (2024) reported that temperature, nitrate, and orthophosphate concentrations affected the microorganisms in the Seine River in the Paris region. Luo et al. (2020) reported that DO, pH, and EC significantly affected the water bacterial communities in the Lancang River. The environmental factors that affected the microbial community structure in the Wuding River Basin differed from those in the abovementioned rivers. We found that elevation affected the microorganisms in the upstream area of the basin, which may have been because the upstream area was a nutrient-poor, high-light environment, and the influence of geographic factors (elevation) on microorganisms was greater than that of nutrients (TOC, COD, and TP, etc.). Downstream, under multiple environmental stresses, temperature and the TP, TOC, and NO_3_-N concentration gradients synergized to drive the microbial community structure differentiation process. Many studies have demonstrated that temperature influences the microbial community structures in rivers (Bagagnan et al., 2024; Gao et al., 2024).

### 3.5 Analysis of microbial carbon cycling and its influencing factors

**FIGURE 7:**
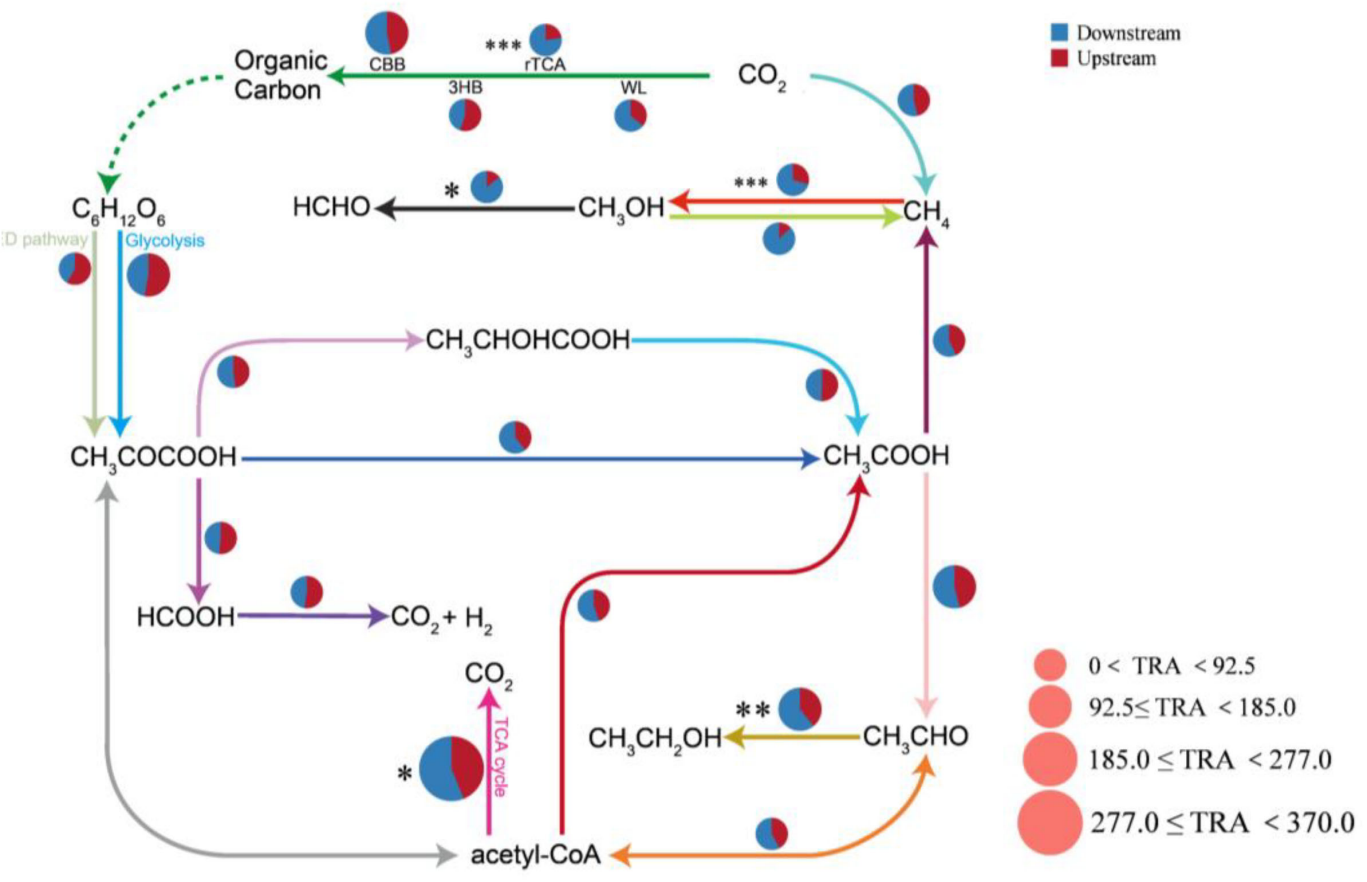
Functional gene pathway analysis of the upstream and downstream carbon cycles in the river. CBB, Calvin-Benson-Bassham cycle; rTCA, reductive citric acid cycle; WL, Wood-Ljungdahl pathway; 3HB, 3-hydroxypropionate bicycle; TRA, the total relative abundance of each pathway (*, *p* < 0.05; **, *p* < 0.01; ***, *p* < 0.001).

By using the KEGG database, we detected a total of 20 carbon cycle reaction pathways, including four carbon sequestration pathways, ten carbon degradation pathways, and six methane metabolism pathways. The TCA cycle pathway had the greatest total relative abundance. The relative abundances of the carbon cycle pathways differed greatly between the upstream and downstream areas of the Wuding River Basin: the downstream area had higher abundances with the methane metabolism pathway (especially the methanol methanogenesis process), the TCA cycle, and the Wood–Ljungdahl pathway (WL, Figure 7). In this study, the relationships between environmental factors and microbial carbon cycle response pathways were also investigated using RDA and Spearman correlation heatmaps (Supplementary Figure 4). The first two principal components of the RDA (RDA1 and RDA2) explained 43.11% and 18.44% of the variance in the functionality of the microbial carbon cycle, respectively (*p* = 0.036). Elevation, TN, NO_3_-N, SS, and COD affected the functional genes for the carbon cycle in the Wuding River Basin, among which TN and NO_3_-N influenced the upstream microbial carbon cycle and TOC, SS, and COD influenced the downstream microbial carbon cycle.

Among the carbon sequestration pathway, Photosystems I and II were significantly positively correlated with elevation (*R* = 0.47, *p* < 0.05) and significantly negatively correlated with TP (*R* = −0.43, *p* < 0.05), NO_3_-N (*R* = −0.49, *p* < 0.05) and SS (*R* = −0.41, *p* = 0.057). The relative abundances of Photosystems I and II were significantly greater upstream than downstream (*p* < 0.05, *t*-test). High elevations usually have stronger solar radiation levels and lower air temperatures, and plants may adapt to this type of environment by increasing their photosystem activity (e.g., the photochemical efficiency of PSII) to maintain their photosynthetic efficiency (Fromme et al., 2006; Li et al., 2023). High concentrations of TP and NO_3_-N may lead to the eutrophication of water bodies or soils and trigger the rapid proliferation of algae or microorganisms, which reduce light and decrease the efficiency of photosystem-driven light energy utilization. SS may adsorb nutrients (e.g., phosphorus and nitrogen) in the water column, alter the bioavailability of the nutrients, and indirectly affect photosystem-driven carbon fixation processes (Luo et al., 2025). The same carbon sequestration pathway, the rTCA cycle, was significantly negatively correlated with elevation (*R* = −0.60, *p* < 0.01) and positively correlated with NH_3_-N (*R* = 0.54, *p* < 0.01), TP (*R* = 0.73, *p* < 0.001), SS (*R* = 0.77, *p* < 0.001), and DOC (*R* = 0.57, *p* < 0.01). The relative abundance of rTCA cycle (*p* < 0.05, *t*-test) was significantly greater downstream than upstream. This occurred because the rTCA cycle pathway is not dependent on light, and the low-elevation downstream environment created suitable conditions for the growth of rTCA cycle microorganisms by enriching nutrients (NH_3_-N, TP, and DOC) and SS (Li et al., 2023; Tang et al., 2011).

For the carbon degradation pathway, the TCA cycle was negatively correlated with elevation (*R* = −0.44, *p* < 0.05) and significantly positively correlated with TN (*R* = 0.49, *p* < 0.05), temperature (*R* = 0.56, *p* < 0.01), NO_3_-N (*R* = 0.45, *p* < 0.05), DOC (*R* = 0.53, *p* < 0.05), and TOC (*R* = 0.43, *p* < 0.05); the relative abundance of the TCA cycle (*p* < 0.05, *t*-test) was significantly greater downstream than upstream. This was attributed to the lower elevation and higher temperature observed downstream, which accelerated the rate of enzymatic reactions and enhanced the metabolic activity of microorganisms; the abundance of the TCA cycle (as a core catabolic pathway) increased significantly with increasing temperature (Chen et al., 2023; Koloti et al., 2024). Elevated concentrations of TOC and DOC provided heterotrophic microorganisms with substrates (e.g., glucose and fatty acids) that entered the TCA cycle through glycolysis to produce pyruvate and increased their abundance (Campbell et al., 2022; Yin et al., 2024).

For the methane metabolic pathway, methane oxidation was significantly positively correlated with TP (*R* = 0.43, *p* < 0.05), SS (*R* = 0.55, *p* < 0.01), DOC (*R* = 0.63, *p* < 0.01), and TOC (*R* = 0.59, *p* < 0.01), and the relative abundance of methane oxidation (*p* < 0.01, *t*-test) was significantly greater downstream than upstream. This is because SS increased the availability of nutrients in the sediment by adsorbing phosphorus and organic matter, which promoted the metabolic activities of methane-oxidizing bacteria (Li et al., 2021; Scanlan et al., 2022). TOC and DOC are decomposed by methanogenic bacteria in anaerobic environments, which generates a large amount of methane and provides substrate for downstream methane oxidation (Ni et al., 2024; Zheng X-C. et al., 2023).

In summary, the indicator species in the upstream region of the watershed were dominated by light-energy autotrophic microbial communities, whereas the downstream region was dominated by heterotrophic, carbon-metabolizing microbial communities. This spatial distribution was consistent with the microbial functional group analysis of the key carbon cycle pathways (Photosystems I and II, the rTCA cycle, the TCA cycle, and methane oxidation). Geomorphic heterogeneity indirectly affected the functional characteristics of the microbial carbon cycle by influencing environmental factors (e.g., TN and COD).

### 3.6 Analysis of microbial nitrogen cycling and its influencing factors

**FIGURE 8:**
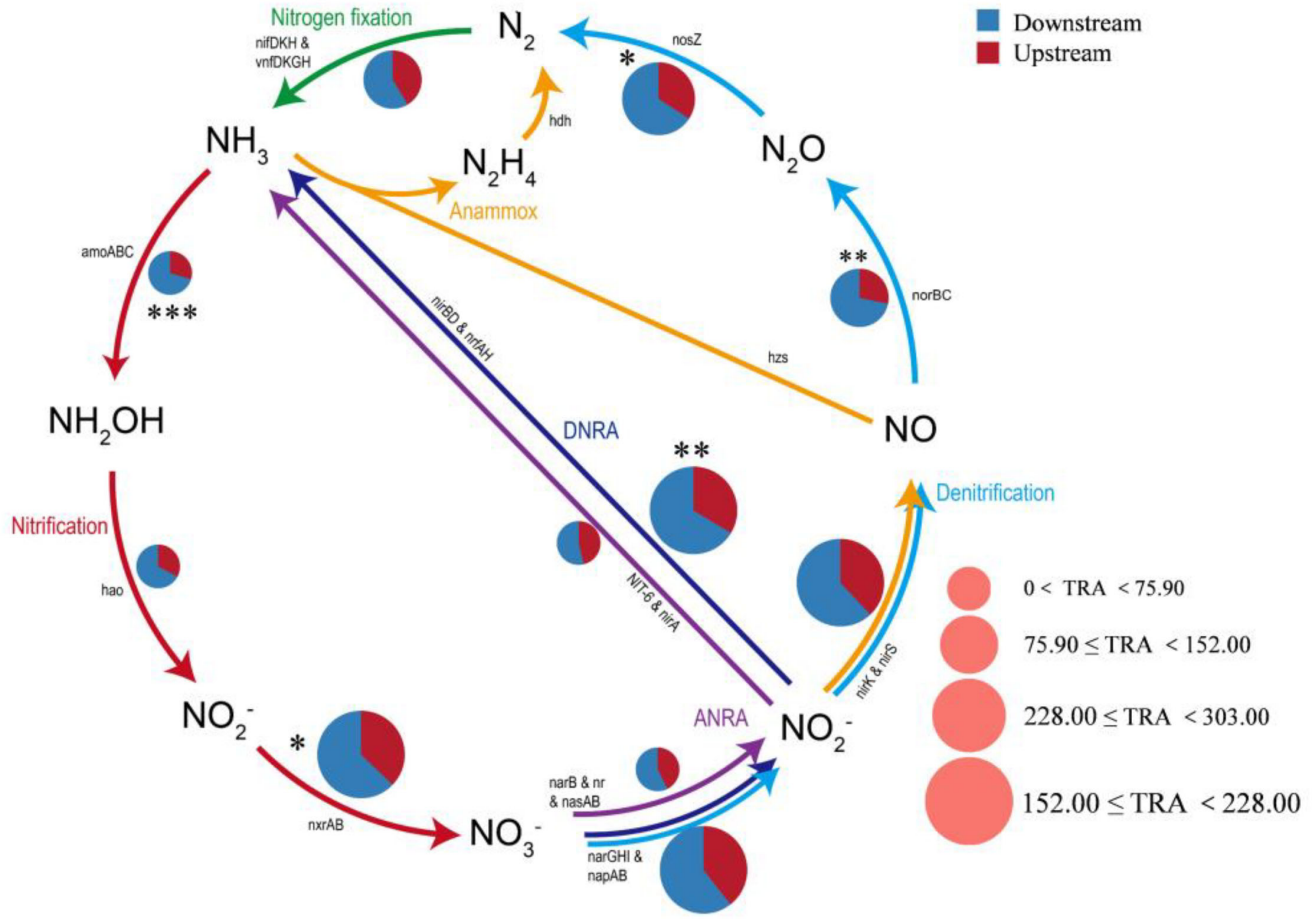
Functional gene pathway analysis of the upstream and downstream nitrogen cycles in the river. ANRA, assimilatory nitrate reduction to ammonium; DNRA, dissimilatory nitrate reduction to ammonium; TRA, the total relative abundance of each pathway (*, *p* < 0.05; **, *p* < 0.01; ***, *p* < 0.001).

A total of five modes of nitrogen metabolism and the related genes were detected using the KEGG annotated gene database (Figure 8), including dissimilatory nitrate reduction to ammonium (DNRA, *narGHI*, *napAB*, *nirBD*, and *nrfAH*), assimilatory nitrate reduction to ammonium (ANRA, *narB*, *NR*, *nanna*, *nit6*, and *nirA*), denitrification (*nirK*, *nirS*, *norBC*, and *nosZ*), nitrogen fixation (*nifKDH*), and nitrification (*amoABC*, *hao*, and *nxrAB*). DNRA had the highest relative abundance (34.00%), followed by denitrification (33.64%). The relative abundance of the genes involved in the rate-limiting step of nitrification (i.e., *amoABC* and *hao*) was generally low (3.22%), whereas the abundance of genes converting NO_2_^–^ to NO_3_^–^ (i.e., *nxrAB*) was relatively high (18.56%). Overall, the abundances of nitrogen-related genes differed significantly between the upstream and downstream regions. The relative abundances of nitrogen-cycle functional genes, including *nosZ* (*p* < 0.05), *norBC* (*p* < 0.01), *amoABC* (*p* < 0.001), *nxrAB* (*p* < 0.05) and *nirBD* (*p* < 0.01), were significantly higher in the downstream region than in the upstream region. The relative abundances of the downstream nitrogen functional genes were generally greater than those of the upstream genes, suggesting that the downstream environment was more competitive and microorganisms responded to changing nutrient conditions through multiple metabolic pathways (Sebastián et al., 2018). In this study, the relationships between environmental factors and microbial nitrogen cycle reaction pathways were also analyzed using RDA and heatmaps. RDA1 (68.17%) and RDA2 (10.6%) together explained 78.77% of the variance, but RDA1 was the main driver (*p* = 0.013). Eutrophication-related factors such as COD, TOC, and NH_3_-N were important regulators of downstream microbial community nitrogen cycling functions, whereas elevation and pH drove upstream microbial nitrogen cycling (Supplementary Figure 5).

From an ecological functional perspective, the distribution of these nitrogen transformation pathways significantly influences nitrogen return and potential eutrophication risks. In our research, the high potential of DNRA is particularly noteworthy, as evidenced by the high abundance of key genes such as *narGHI*, *napAB*, and *nrfAH*. DNRA reduces NO_3_ to NH_4_^+^, thereby retaining nitrogen within the ecosystem rather than removing it directly (Smith et al., 2007). This process may enhance the watershed’s nitrogen retention capacity. Coupled with elevated concentrations of TOC and COD in downstream areas, this enhanced DNRA is likely facilitated by TOC-driven reducing conditions. Whilst this process retains nitrogen, it also increases the risk of sustained endogenous release of bioavailable nitrogen (in ammonium form), potentially elevating eutrophication potential (Li et al., 2019; Nogaro and Burgin, 2014).

On the other hand, denitrification serves as the primary pathway for nitrogen removal, reducing nitrate to gaseous nitrogen (N_2_ or N_2_O), resulting in the permanent removal of nitrogen from aquatic systems (Gao et al., 2021). Although the overall abundance of denitrifying genes is relatively high (33.64%), its efficiency is strongly regulated by environmental conditions (such as DO concentration and organic carbon availability). In downstream areas, despite high gene abundance, the process may prove incomplete if denitrification activity is suppressed by hydrological disturbances or unsuitable carbon source composition. This may lead to the accumulation of intermediate products, such as the potent greenhouse gas N_2_O, or reduced denitrification efficiency, thereby indirectly exacerbating nitrogen accumulation (Nevorski and Marcarelli, 2022).

A low total abundance for the microorganisms involved in the first step of nitrification (the nitritation process) suggests low activity in the corresponding functional pathways (*amoABC* and *hao*) and limited NO_2_^–^-N production. However, the NO_2_^–^-N produced by the other pathways (ANRA, DNRA, and denitrification) can still be used as a substrate for nitrification to produce NO_3_^–^-N. In addition, the NO_3_^–^-N concentration increased with increasing *narGHI* and *napAB* abundance. This result is consistent with the findings of Jiang et al. (2023). The microbial genera with higher relative abundances were *Pseudomonas* and *Hydrogenophaga*, and the involvement of these genera in DNRA may explain the relatively high relative abundances of DNRA genes in the nitrogen cycle (Kelly et al., 2021). Moreover, *Pseudomonas* (*p* = 0.027, *t*-test) and *Hydrogenophaga (p* = 0.017, *t*-test) presented significantly greater relative abundances downstream than upstream, and *Pseudomonas* served as a bioindicator in the downstream area. This likely explains why the relative abundance of microorganisms performing DNRA was greater downstream than upstream. During the nitrogen cycle, denitrification was significantly less efficient than the other nitrogen transformation pathways (e.g., nitrification and ammonification) in terms of removing nitrogen, and insufficient functional gene abundance and metabolic activity may lead to nitrogen retention in the water.

In addition, some microbial nitrogen cycling pathways in the Wuding River Basin were significantly correlated with SS, TP, NH_3_-N, NO_3_-N, DO, TOC, DOC, and elevation. SS forms a particulate microenvironment through the adsorption of organic matter, nutrients, and microorganisms, and there is often a gradient of oxygen on the surface (surface aerobic, internal anoxic) that provides the reaction conditions for nitrogen cycling (de Beer et al., 1994). Phosphorus is an essential element for ATP, nucleic acid, and cell membrane synthesis, and elevated TP concentrations can alleviate phosphorus limitations, promote the uptake of nitrogen by phytoplankton, and indirectly accelerate the nitrogen assimilation and recirculation processes (Chtouki et al., 2024; Hessen, 2013). TOC and DOC affect the nitrogen cycle by influencing microbial metabolism, providing an energy source, and participating in substance transformation (Gihring et al., 2010). Overall, the microbial nitrogen cycling pathways in the Wuding River Basin involve a complex network of interactions and multidimensional environmental factors.

## 4 Conclusion

This research reveals the heterogeneity in functional potential within the carbon and nitrogen cycles, as well as in water quality and microbial community composition, between the upstream and downstream regions of the Wuding River basin, driven by differing geological and topographical conditions. Our conclusions are summarized below:

1. The Wuding River exhibited significant spatial water quality heterogeneity, which was driven by geographic, geomorphic, and anthropogenic factors. Compared with the upstream regions, the downstream regions presented significantly higher temperature (*p* < 0.01), TOC (*p* < 0.001), DOC (*p* < 0.001), COD (*p* < 0.05), and TP (*p* < 0.001) levels.

2. Microbial alpha and beta diversity displayed upstream–downstream divergence. The downstream areas presented significantly greater ACE (*p* < 0.05), Chao1 (*p* < 0.05), Shannon (*p* < 0.001), and Pielou’s evenness (*p* < 0.001) indices. Elevation drove the upstream alpha diversity, whereas temperature, TP, and NO_3_-N influenced the downstream alpha diversity.

3. *Pseudomonadota*, *Cyanobacteriota*, and *Actinomycetota* dominated the microbial communities. An LEfSe analysis revealed that upstream communities were adapted to oligotrophic, high-light environments, whereas downstream communities were associated primarily with complex heterotrophic carbon metabolism. The environmental drivers varied: elevation shaped the upstream microbial communities, whereas temperature, TP, TOC, and NO3-N influenced the downstream microbial communities.

4. The downstream region of the river basin presented increased carbon cycling (e.g., methane metabolism and the TCA/rTCA cycle) and nitrogen functional gene abundances. Upstream carbon cycling was regulated by TN and NO_3_-N, and nitrogen cycling responded to elevation and pH Conversely, the downstream carbon and nitrogen cycles were controlled by TOC, SS, COD, and NH_3_-N.

The research findings provide a theoretical basis and practical pathway for the precise management of water quality and ecological restoration within the Wuding River basin. Based on differences in upstream and downstream environmental characteristics and microbial functions, we recommend implementing a “zoned management” strategy. In upstream areas (the Mu Us Landsand), we should prioritize water conservation and ecological protection, restricting excessive human interference; in the downstream (Loess Plateau region), we must focus on controlling external pollution inputs and addressing eutrophication, with particular emphasis on intercepting agricultural non-point source pollution and enhancing urban sewage treatment. In the future research, we should systematically investigate the mechanisms by which microbial community structures respond to changes in aquatic ecosystems across multiple seasons and years. We should further establish a river health diagnosis and early warning system, thereby advancing watershed management from a focus on “water quality targets” toward “ecological function targets.”

## Data Availability

The data presented in the study are deposited in the Genome Sequence Archive (GSA) repository, accession number CRA032658.

## Footnotes

1
https://www.kegg.jp/
