# Supplementary material for: Factors influencing the spatial distributions of river microbial communities at the watershed scale: a case study involving the Wuding River Basin
Source: Front Microbiol. 2025 Nov 11;16:1667966. doi: 10.3389/fmicb.2025.1667966 (PMC12646543; doi:10.3389/fmicb.2025.1667966)
Supplement: Supplementary file 1 [file Supplementary_file_1.docx]

**Factors influencing the spatial distributions of river microbial communities at the watershed scale: a case study involving the Wuding River Basin**

Nan Xue^1,2,3†^, Manhong Xia^1,2,3†^, Bo Hu^4^, Xinru Gong^4^, Zhoufeng Wang^1,2,3*^ and Xiaohong Zhao^1,4*^

^1^ Key Laboratory of Subsurface Hydrology and Ecological Effect in Arid Region of the Ministry of Education, Chang’an University, Xi’an, China

^2^ School of Water and Environment, Chang’an University, Xi’an, China

^3^ Key Laboratory of Eco-hydrology and Water Security in Arid and Semi-arid Regions of Ministry of Water Resources, Chang’an University, Xi’an, China

^4^ School of Civil Engineering, Chang’an University, Xi’an, China

****Co-first author:**

Nan Xue and Manhong Xia contributed equally to this manuscript.

***Corresponding author:**

E-mail address: [wangzf@chd.edu.cn](mailto:wangzf@chd.edu.cn) (Z. Wang); [xzhao@chd.edu.cn](mailto:xzhao@chd.edu.cn) (X. Zhao)

# List of text and table captions

**Table S1** Hydrogeochemical parameters upstream and downstream of the Wuding River Basin

**Text S1** Chemical oxygen demand (COD) sampling method

**Fig. S1.** Analysis of physico-chemical properties of water bodies upstream and downstream of the Wuding River Basin (***, *p* < 0.001)

**Fig. S2.** Analysis of microbial beta diversity in the Wuding River Basin (NMDS (A) and PCoA (B) analyses based on Bray-Curtis distance)

**Fig. S3.** Linear discriminant analysis effect size (LEfSe) analysis of water microbial communities in upstream and downstream samples. Cladogram showing the phylogenetic structure of the microbiota. In the branching diagram of evolution, circles radiating from inside to outside represent the taxonomic level from boundary to species, and each small circle at different taxonomic levels represents a species at the taxonomic level.

**Fig. S4.** Environmental factors affecting microbial carbon cycling in the Wuding River Basin. (A) RDA analysis at genus level; (B) Spearman correlation heat map analysis of environmental factors and carbon cycle pathways.

**Fig. S5.** Environmental factors affecting microbial nitrogen cycling in the Wuding River Basin. (A) RDA analysis at genus level; (B) Spearman correlation heat map analysis of environmental factors and carbon cycle pathways.

**Table S1** Hydrogeochemical parameters upstream and downstream of the Wuding River Basin

| group | Sample | Elevation | Temp | pH | DO | EC | NH_3_-N | NO_3_-N | TN | TP | COD | SS | DOC | TOC |
| --- | --- | --- | --- | --- | --- | --- | --- | --- | --- | --- | --- | --- | --- | --- |
|  |  | m | ℃ |  | % | uS/cm | mg/L | mg/L | mg/L | mg/L | mg/L | mg/L | mg/L | mg/L |
| Downstream | WSW1 | 665.5 | 24.3 | 8.07 | 95.7 | 781 | 0.96 | 1.78 | 10.45 | 1.44 | 11.98 | 4482 | 9.4 | 12.4 |
|  | WSW2 | 792.8 | 22.1 | 8.06 | 75.2 | 978 | 1.38 | 1.89 | 7.3 | 2.94 | 82.75 | 7668 | 8.4 | 12.4 |
|  | WSW3 | 829.7 | 23.2 | 8.04 | 85.9 | 1183 | 1.64 | 0.26 | 6.75 | 2.94 | 111.79 | 11356 | 9.1 | 10.9 |
|  | WSW4 | 850.5 | 24.7 | 8.15 | 89 | 974 | 0.32 | 2.24 | 9.95 | 0.84 | 17.64 | 43674 | 13.4 | 14.1 |
|  | WSW5 | 960.8 | 24.9 | 8.18 | 74.5 | 948 | 0.18 | 2.16 | 5.9 | 0.36 | 45.37 | 1224 | 15.9 | 16.5 |
|  | WSW6 | 1025.9 | 23.9 | 8.32 | 86.4 | 785 | 0.63 | 2.34 | 5.8 | 0.94 | 7.39 | 1006 | 9.5 | 10.9 |
|  | WSW7 | 921.6 | 22.7 | 8.01 | 69 | 1163 | 1.18 | 0.68 | 5.5 | 0.42 | 90.73 | 247056 | 10 | 11.2 |
|  | WSW8 | 879.4 | 23.1 | 8.2 | 74.7 | 811 | 1.41 | 1.82 | 11.35 | 2.76 | 39.86 | 7315 | 7.4 | 9.6 |
|  | WSW9 | 969.2 | 26.4 | 8.03 | 85.4 | 1148 | 1.97 | 1.17 | 8.6 | 1.72 | 49.99 | 53674 | 10.6 | 10.3 |
|  | WSW10 | 898.3 | 26.8 | 8.4 | 73.5 | 755 | 3.3 | 1.31 | 7 | 3.1 | 25.48 | 128820 | 8.5 | 9.9 |
|  | WSW11 | 918.6 | 25.3 | 8.44 | 81.8 | 569 | 0.65 | 2.19 | 5.55 | 1.26 | 18.04 | 6865 | 8.2 | 8 |
| Upstream | WSW12 | 946.2 | 24.4 | 8.3 | 82.7 | 461 | 2.19 | 2.32 | 4.75 | 1.06 | 11.73 | 2030 | 6.3 | 5.9 |
|  | WSW13 | 1069.2 | 23.1 | 8.28 | 85.2 | 616 | 0.67 | 0.45 | 4.5 | 0.12 | 21.47 | 19 | 7.4 | 7 |
|  | WSW14 | 1177.2 | 19.1 | 8.02 | 71.3 | 741 | 0.14 | 0.91 | 4.28 | 0.08 | 25.2 | 7 | 4.3 | 5 |
|  | WSW15 | 940.9 | 22.8 | 8.14 | 71.6 | 975 | 1.02 | 1.14 | 11.45 | 0.6 | 23.29 | 987 | 5.7 | 5.7 |
|  | WSW16 | 989.3 | 23.4 | 8.17 | 78.8 | 986 | 0.11 | 1.62 | 11.8 | 0.24 | 39.86 | 442 | 5 | 5.3 |
|  | WSW17 | 1142.1 | 21.5 | 8.28 | 72.8 | 1666 | 0.55 | 1.67 | 9.3 | 0.12 | 11.95 | 113 | 5 | 5.3 |
|  | WSW18 | 1059.9 | 20.8 | 8.27 | 82.1 | 924 | 0.23 | 2.48 | 5.8 | 0.16 | 20.4 | 316 | 3.6 | 3.4 |
|  | WSW19 | 1009.7 | 16.2 | 8.2 | 86.9 | 692 | 0.11 | 0.25 | 11 | 0.06 | 15.28 | 5 | 4.7 | 7.4 |
|  | WSW20 | 1003.8 | 23.4 | 8.16 | 64 | 1482 | 2.48 | 1.99 | 11.5 | 0.28 | 26.22 | 1000 | 6.6 | 6.5 |
|  | WSW21 | 1117.4 | 19.7 | 8.26 | 63.6 | 723 | 0.23 | 0.63 | 5 | 0.08 | 16.17 | 12 | 5.3 | 5.1 |
|  | WSW22 | 1122.9 | 22.4 | 8.75 | 57.7 | 1167 | 0.39 | 0.07 | 4.63 | 0.08 | 32.26 | 24 | 6.3 | 6.8 |

Note: Temp: Temperature; DO: Dissolved Oxygen; EC: Electrical Conductivity; NH_3_-N: Ammonia Nitrogen; NO_3_-N: Nitrate Nitrogen; TN: Total Nitrogen; TP: Total Phosphorus; COD: Chemical Oxygen Demand; SS: Suspended Sediment; DOC: Dissolved Organic Carbon; TOC: Total Organic Carbon.

**Text S1**:

The principle and procedure of this standard method are as follows: First, chemical digestion is carried out. The water sample is mixed with a known amount of potassium dichromate digestion solution (containing a sulfuric acid-silver sulfate catalyst) in a sealed digestion tube and undergoes complete chemical oxidation at a high temperature (165 °C) for approximately 15 minutes. This step shares the identical chemical principle with the traditional reflux titration method (e.g., HJ 828-2017), ensuring the thorough oxidation of organic matter. After digestion, the remaining dichromate ions (Cr_2_O_7_^2-^) in the reaction solution appear yellow, while the reduced trivalent chromium ions (Cr^3+^) appear bluish-green. The role of the DR6000 UV-Visible spectrophotometer is to measure the absorbance of the post-reaction mixture at a specific wavelength. This absorbance value is proportional to the concentration of the remaining potassium dichromate, thereby allowing for the calculation of the amount of dichromate consumed and, subsequently, the COD value of the water sample.


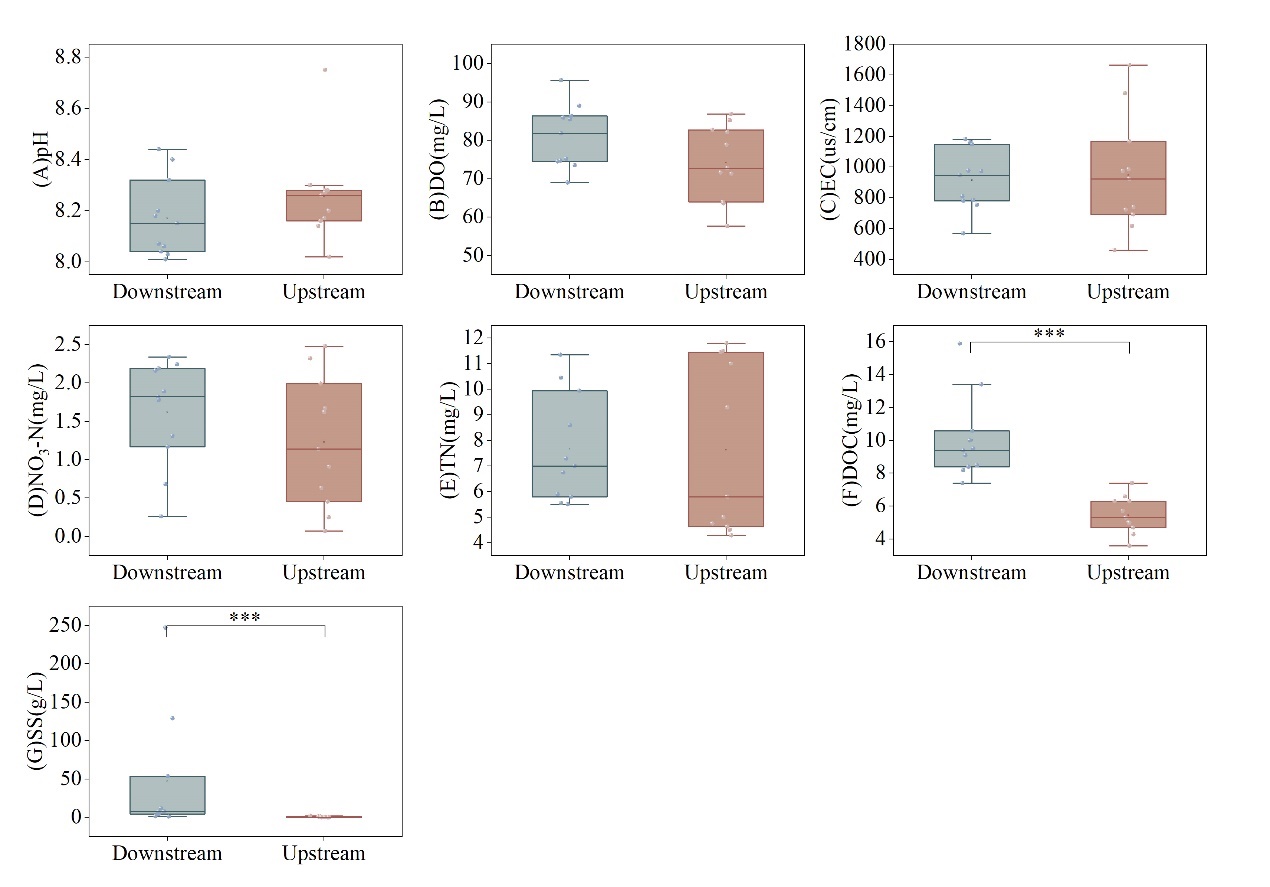


**Fig. S1.** Analysis of physico-chemical properties of water bodies upstream and downstream of the Wuding River Basin (***, p < 0.001)


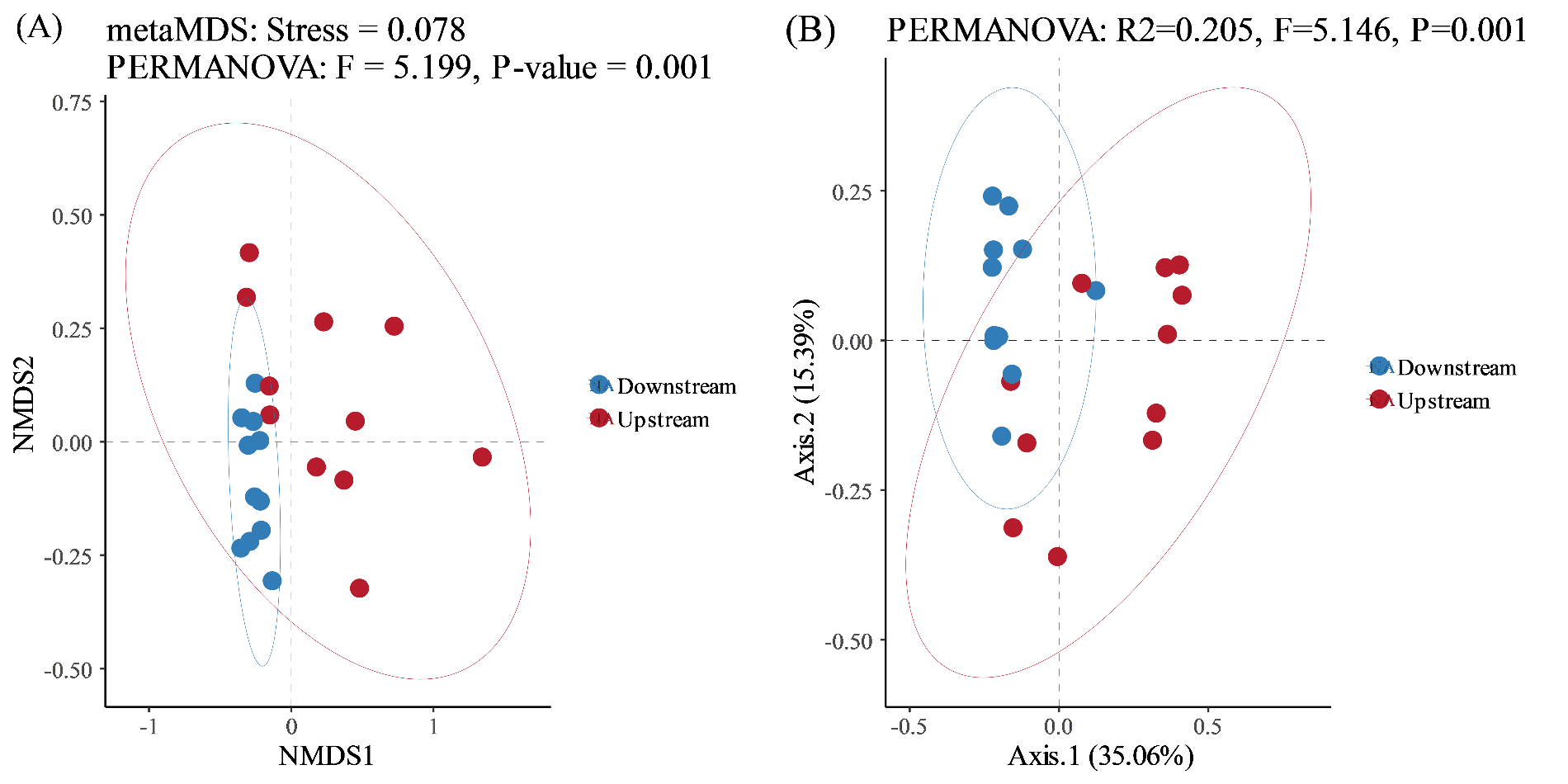


**Fig. S2.** Analysis of microbial β-diversity in the Wuding River Basin (NMDS (A) and PCoA (B) analyses based on Bray-Curtis distance)

**
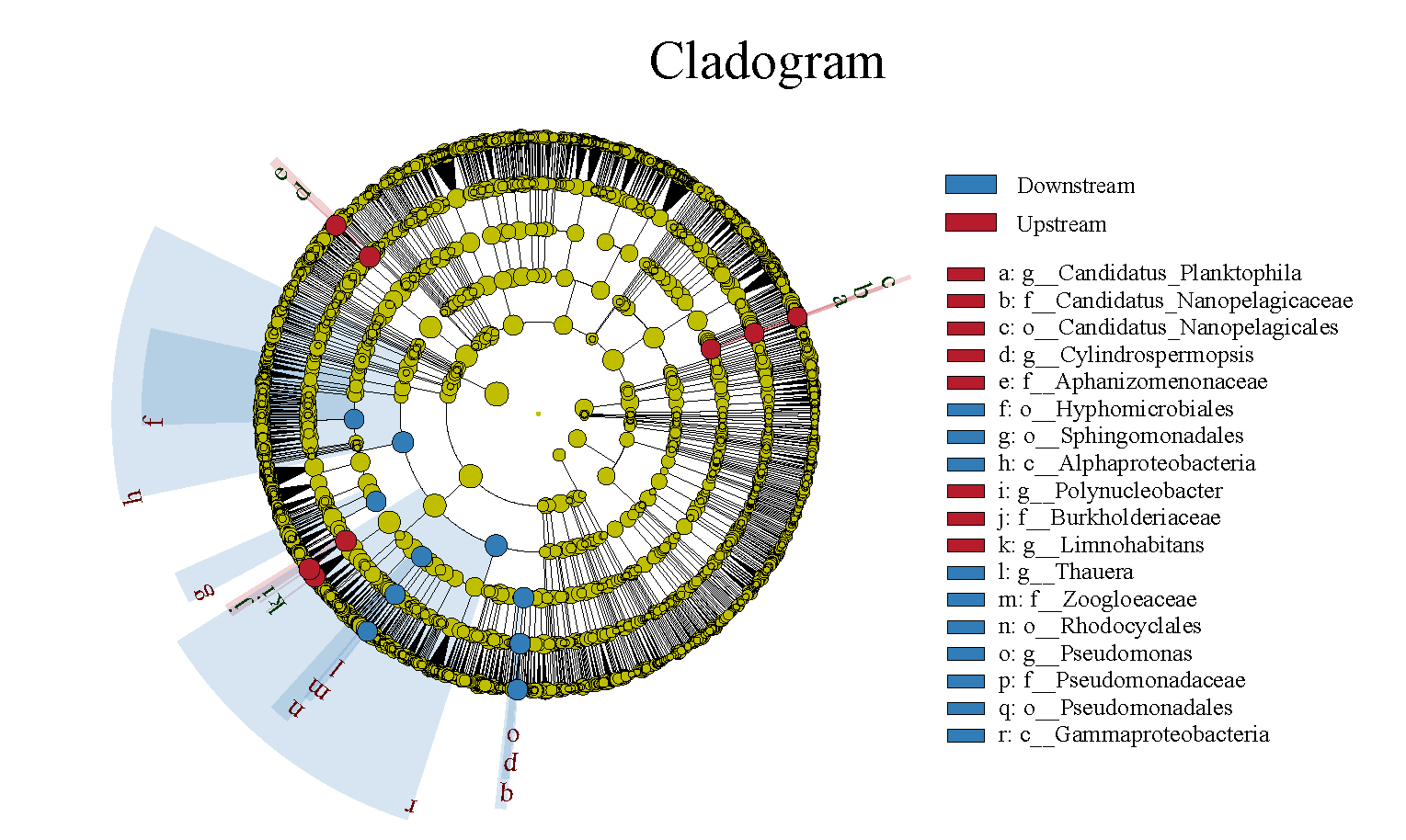
**

**Fig. S3.** Linear discriminant analysis effect size (LEfSe) analysis of water microbial communities in upstream and downstream samples. Cladogram showing the phylogenetic structure of the microbiota. In the branching diagram of evolution, circles radiating from inside to outside represent the taxonomic level from boundary to species, and each small circle at different taxonomic levels represents a species at the taxonomic level.


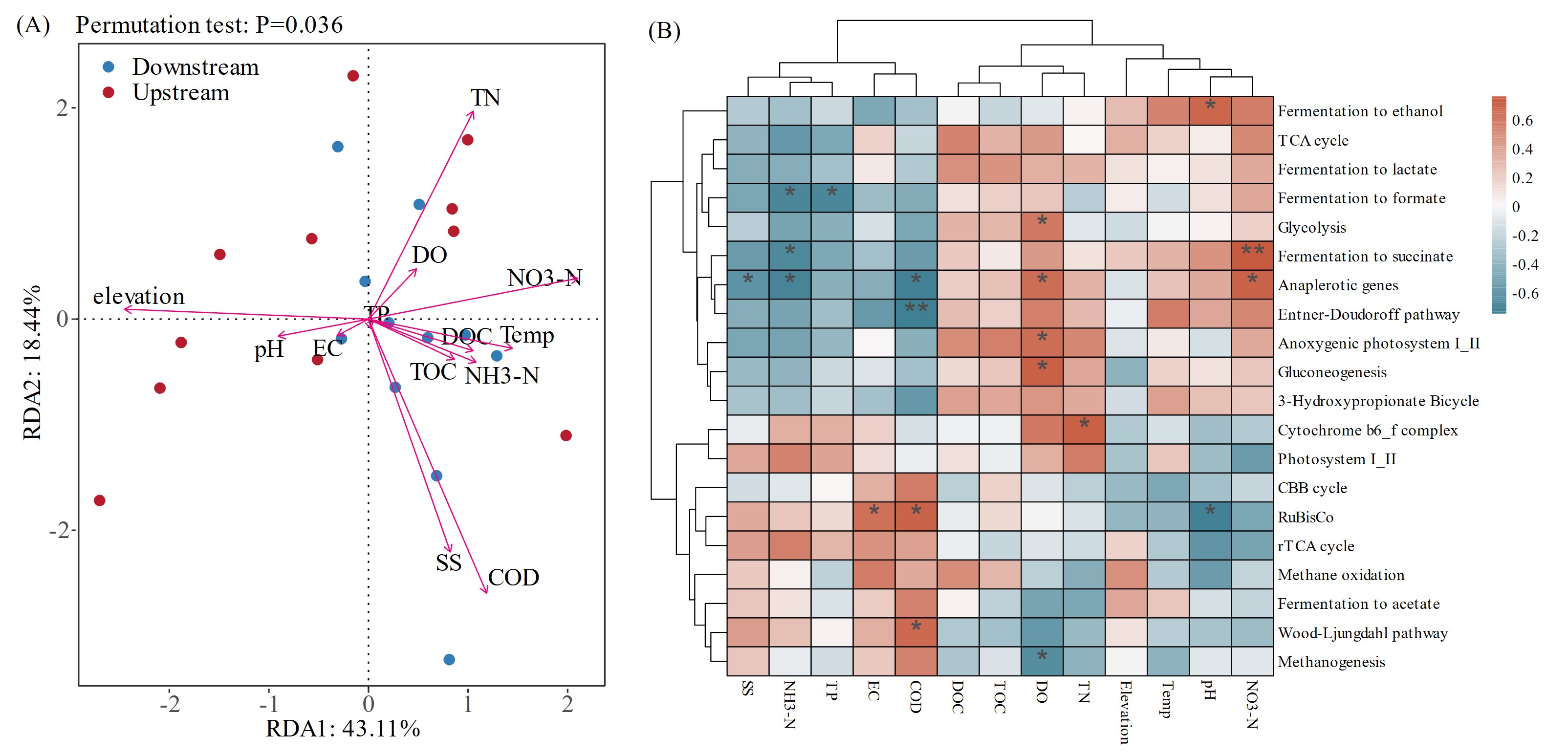


**Fig. S4.** Environmental factors affecting microbial carbon cycling in the Wuding River Basin. (A) RDA analysis at genus level; (B) Spearman correlation heat map analysis of environmental factors and carbon cycle pathways.


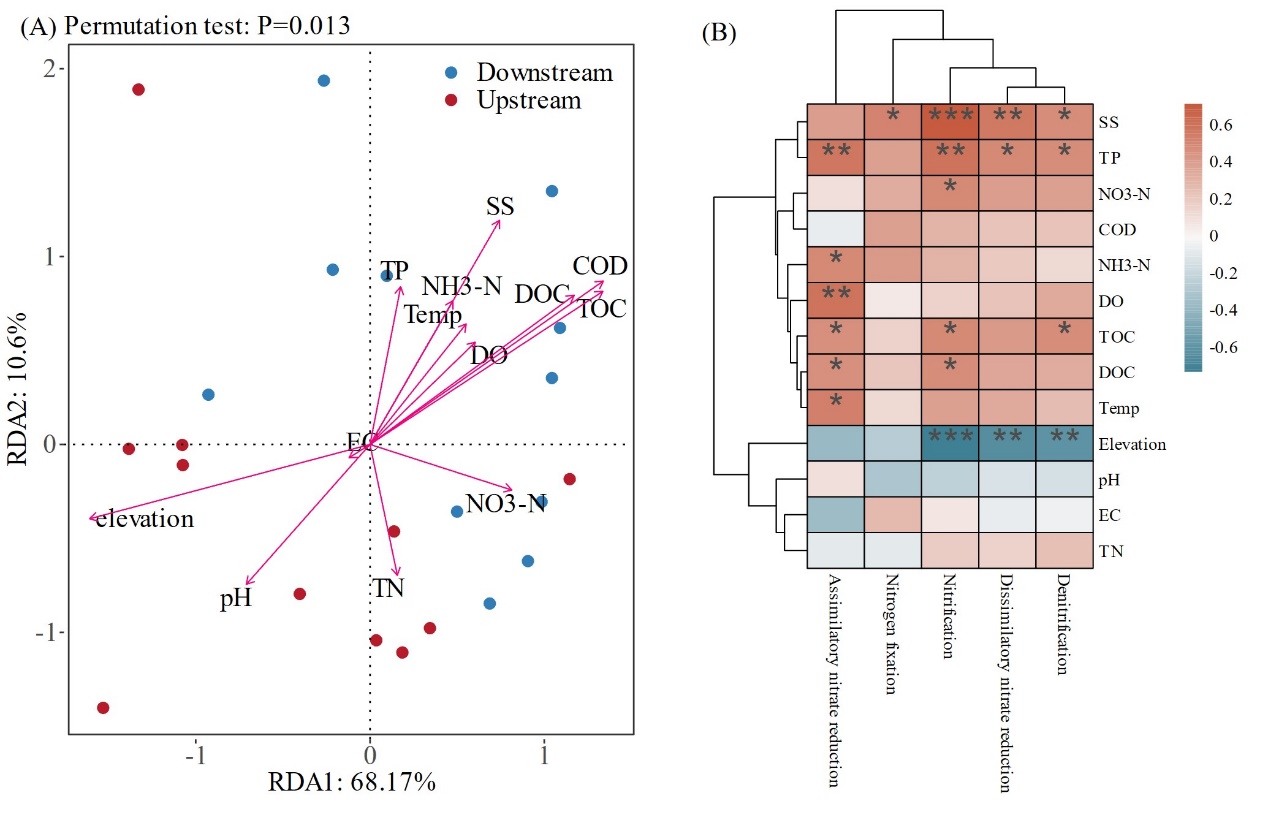


**Fig. S5.** Environmental factors affecting microbial nitrogen cycling in the Wuding River Basin. (A) RDA analysis at genus level; (B) Spearman correlation heat map analysis of environmental factors and carbon cycle pathways.
